# Design of multi-epitope peptides containing HLA class-I and class-II-restricted epitopes derived from immunogenic *Leishmania* proteins, and evaluation of CD4+ and CD8+ T cell responses induced in cured cutaneous leishmaniasis subjects

**DOI:** 10.1371/journal.pntd.0008093

**Published:** 2020-03-16

**Authors:** Sarra Hamrouni, Rachel Bras-Gonçalves, Abdelhamid Kidar, Karim Aoun, Rym Chamakh-Ayari, Elodie Petitdidier, Yasmine Messaoudi, Julie Pagniez, Jean-Loup Lemesre, Amel Meddeb-Garnaoui

**Affiliations:** 1 Laboratoire de Parasitologie Médicale, Biotechnologie et Biomolécules, Institut Pasteur de Tunis, Tunis, Tunisie; 2 Faculté des Sciences de Bizerte, Université de Carthage, Tunis, Tunisie; 3 UMR INTERTRYP, Université de Montpellier, IRD, CIRAD, Montpellier, France; 4 Hôpital Régional de Gafsa, Gafsa, Tunisia; University of Notre Dame, UNITED STATES

## Abstract

Human leishmaniasis is a public health problem worldwide for which the development of a vaccine remains a challenge. T cell-mediated immune responses are crucial for protection. Peptide vaccines based on the identification of immunodominant T cell epitopes able to induce T cell specific immune responses constitute a promising strategy. Here, we report the identification of human leukocyte antigen class-I (HLA-I) and -II (HLA-II)-restricted multi-epitope peptides from *Leishmania* proteins that we have previously described as vaccine candidates. Promastigote Surface Antigen (PSA), LmlRAB (*L*. *major* large RAB GTPase) and Histone (H2B) were screened, *in silico*, for T cell epitopes. 6 HLA-I and 5 HLA-II-restricted multi-epitope peptides, able to bind to the most frequent HLA molecules, were designed and used as pools to stimulate PBMCs from individuals with healed cutaneous leishmaniasis. IFN-γ, IL-10, TNF-α and granzyme B (GrB) production was evaluated by ELISA/CBA. The frequency of IFN-γ-producing T cells was quantified by ELISpot. T cells secreting cytokines and memory T cells were analyzed by flow cytometry. 16 of 25 peptide pools containing HLA-I, HLA-II or HLA-I and -II peptides were able to induce specific and significant IFN-γ levels. No IL-10 was detected. 6 peptide pools were selected among those inducing the highest IFN-γ levels for further characterization. 3/6 pools were able to induce a significant increase of the percentages of CD4+IFN-γ+, CD8+IFN-γ+ and CD4+GrB+ T cells. The same pools also induced a significant increase of the percentages of bifunctional IFN-γ+/TNF-α+CD4+ and/or central memory T cells. We identified highly promiscuous HLA-I and -II restricted epitope combinations from H2B, PSA and LmlRAB proteins that stimulate both CD4+ and CD8+ T cell responses in recovered individuals. These multi-epitope peptides could be used as potential components of a polytope vaccine for human leishmaniasis.

## Author summary

The control of leishmaniasis, a neglected tropical disease of public health importance, caused by protozoan parasites of the genus *Leishmania*, mainly relies on chemotherapy, which is highly toxic. Currently, there is no vaccine against human leishmaniasis. Peptide-based vaccines consisting of T cell epitopes identified within proteins of interest by epitope predictive algorithms are a promising strategy for vaccine development. Here, we identified multi-epitope peptides composed of HLA-I and -II-restricted epitopes, using immunoinformatic tools, within *Leishmania* proteins previously described as potential vaccine candidates. We showed that multi-epitope peptides used as pools were able to activate IFN-γ producing CD4+ as well as CD8+ T cells, both required for parasite elimination. In addition, granzyme B-producing CD4+ T cells, bifunctional CD4+ IFN-γ+/TNF-α+ and/or TNF-α+/IL-2+ T cells as well as CD4+ and CD8+ central memory T cells, all involved in *Leishmania* infection control, were significantly increased in response to multi-epitope peptide stimulation. As far as we know, no study has described the detection of both CD4+ and CD8+ T cell populations in response to stimulation by both HLA-I and II-restricted peptides in humans. The immunogenic HLA-I and -II-restricted multi-epitope peptides identified in this study could constitute potential vaccine candidates against human leishmaniasis.

## Introduction

Leishmaniasis is caused by an intracellular parasite of the *Leishmania* genus. It is a severely neglected tropical disease associated with considerable morbidity and mortality throughout the world. This disease transmitted by sand fly bites can have a wide spectrum of clinical manifestations ranging from self-healing cutaneous lesions to fatal visceral disease, depending on the infecting parasite species, the host immune response and the sand fly saliva components [1, 2]. Cutaneous leishmaniasis (CL) is the most frequent form with 0.7–1 million new cases occurring annually worldwide [1, 3]. Zoonotic CL, caused by *Leishmania* (*L*.) *major*, is endemic in Tunisia, with thousands of cases reported every year [4, 5]. The control of leishmaniasis mainly relies on chemotherapy, which is highly toxic and contributes to the emergence of drug-resistant parasites [6]. Most individuals who recover from leishmaniasis develop lifelong immunity to re-infection suggesting that a vaccine is feasible. It is generally considered that a dominant T helper 1 (Th1)-type cellular immune response and IFN-γ production are critical for infection control. In the murine model of *L*. *major* infection, CD4+ Th1 cells secrete IFN-γ and TNF-α, leading to the parasite elimination by activated macrophages, whereas, CD4+ Th2 response producing IL-4 and IL-13 favors disease progression [7, 8]. Th1/Th2 dichotomy is absent in human leishmaniasis. It is now clear that the immune response against *Leishmania* parasites is more complex both in humans and mice [9]. In human infection, Th1 CD4+ T cells producing IFN-γ and TNF-α and positive delayed type hypersensitivity (DTH) responses, have been associated with the healing process [10–14]. IL-10 was associated with a lack of parasite control but may also play a role in the control of excessive inflammatory response [15–19]. The induction of multifunctional Th1 cells secreting IFN-γ, TNF-α and IL-2, has been described to correlate with protection [20–23]. CD8+ T cells are also important in the healing mainly through IFN-γ production [24–26]. These cells have also been involved in pathogenesis, trough Granzyme B (GrB) production [27–30], while other studies showed that an increase of GrB activity was associated with a good prognosis in patients with CL [31–33]. Both central (TCM) and effector memory T cells (TEM) have been characterized in human CL and could play a role in protection against *Leishmania* infection [34–36]

Several vaccination strategies against leishmaniasis have been examined so far including leishmanization, killed or attenuated parasites, DNA and subunits vaccines including native or recombinant proteins [37, 38]. However, there is currently no vaccine for humans. In recent years, the use of peptides containing the minimal immunogenic part of a protein capable of inducing a desired specific T cell response may become a promising strategy in leishmaniasis prophylaxis [39–41]. In addition, the development of bioinformatic tools has made it easier to identify potential immunogenic Human Leukocyte Antigens (HLA)-restricted T cell epitopes for vaccines. Peptide-based vaccines have many advantages including absence of infectious materials, stability, specificity and large-scale production at low cost. Peptide-based vaccines have been successfully tested against cancer and infectious diseases [42–46]. Potential immunogenic peptides can be identified within proteins that have been previously described as vaccine candidates. We already reported that *Leishmania* histone H2B, Promastigote Surface Antigen (PSA) and *L*. *major* large RAB GTPase (LmlRAB), were able to induce a predominant Th1 response in individuals immune to *L*. *major* or *L*. *infantum* [47–49]. These proteins are involved in the organization and function of DNA within the nucleus for histone, and in resistance to complement lysis and macrophage invasion for PSA, whereas a role in phagosome maturation and regulation of the secretory pathway was described for RAB proteins in *Leishmania* parasites. H2B, PSA and LmlRAB are highly conserved among *Leishmania* species while displaying low level of homology with mammalian homologues. They are present in both amastigote and promastigote stages and confer significant protection in mice and dogs [47–52].

Here, we identify potential immunogenic multi-epitope peptides from H2B, PSA and LmlRAB *Leishmania* proteins. Promiscuous T cell epitopes able to bind to several HLA class I (HLA-I) or HLA class II (HLA-II) molecules were identified using online epitope prediction software tools and designed to construct multi-epitope peptides. Their capacity to stimulate cytokine-producing CD4, CD8, multifunctional and memory T cells was evaluated, *in vitro*, in individuals with healed CL due to *L*. *major*.

## Materials and methods

### Ethics statement

The recruitment and sampling collection of groups of volunteers were conducted with the approval of the local ethical committee of the Pasteur Institute of Tunis (protocol number 2015/01/I/LR11IPT06). All subjects analyzed were over 18 years old. A written informed consent was obtained from all subjects before enrollment.

### Study population

Human groups included cured CL and healthy individuals (**Table 1**). Cured individuals were recruited from well-characterized endemic areas for CL due to *L*. *major* located in central Tunisia (Kairouan and Gafsa), based on the following inclusion criteria (i) living in endemic foci to *L*. *major* (ii) well documented medical records (iii) presence of typical scars and (iv) positive IFN-γ response to Soluble *Leishmania* Antigens (SLA). All patients have recovered after a course of standard Glucantime therapy, with the exception of 3 individuals who have practiced self-medication. Healthy individuals were recruited in a low endemic area (Tunis), with no history of leishmaniasis and no IFN-γ response to SLA. Exclusion criteria were immunosuppressive diseases other than leishmaniasis, long-term treatment and pregnancy. Heparinized blood was collected from cured CL donors and healthy individuals used as a control group (**Table 1**).

**Table 1:** Study population.

Status	Cured from CL	Healthy
Number of individuals	61	28
Average age (year) (Mean±SD)	44.6±14.5	32±11.7
Sex ratio (M/F)	0.48	0.3
Average LCZ scar number (Mean±SD)	1.8±1.1	0
IFN-γ response to SLA (pg/ml)*	2298±1689.9/219.9±843	18.9±19.5/64.9±137.7

*Mean of SLA ± SD/Mean of unstimulated ± SD

### HLA typing

DNA was extracted from peripheral blood using QIAamp DNA Blood Mini Kit (QIAGEN GmbH, Germany) according to the manufacturer’s instructions. HLA genotyping of DNA samples was performed by Next Generation Sequencing (NGS) (DKMS Life Science Lab, Dresden, Germany).

### Protein selection

For this study, the full sequences of H2B, LmlRAB and PSA proteins derived from multiple *Leishmania* species (*L*. *major*, *L*. *infantum*, *L*. *donovani*, *L*. *braziliensis*, *L*. *mexicana*, *L*. *amazonensis*, *L*. *chagasi*) were obtained from the national center for biotechnology (NCBI) protein database (http://www.ncbi.nlm.nih.gov/protein/) (**Table 2**).

**Table 2:** *Leishmania* proteins used as candidate antigens for epitope screening.

Abbreviations	Names	*Leishmania* species	Accession number*	AA** number
**H2B**	**Histone**	*L*. *major*	AF336276	111
		*L*. *donovani*	XP_003858819	111
		*L*. *infantum*	XP_001463598	111
		*L*. *braziliensis*	XP_001564132	111
		*L*. *mexicana*	XP_003874182	107
**PSA**	**Promastigote Surface Antigen**	*L*. *amazonensis*	ACY70940	371
		*L*. *infantum*	ACY70941	463
		*L*. *donovani*	AAY96325	386
		*L*. *major*	AAB38549	385
		*L*. *tropica*	AAF80491	626
		*L*. *braziliensis*	XP_001563125	912
		*L*. *mexicana*	XP_003873184	367
		*L*. *chagasi*	AAB62271	417
**LmlRAB**	***Leishmania major* large RAB GTPase**	*L*. *major*	AY962589	611
		*L*. *donovani*	XP_003863179	609
		*L*. *infantum*	XP_001467349	609
		*L*. *braziliensis*	XP_001567099.1	609
		*L*. *mexicana*	XP_003877608.1	610

*The full sequences of the proteins were extracted from NCBI database

**AA: amino acid

Align Sequences Protein BLAST (https://blast.ncbi.nlm.nih.gov/BlastAlign.cgi) and Clustal Omega, a multiple sequence alignment program (https://www.ebi.ac.uk/Tools/msa/clustalo/), were used to analyze sequence similarity between proteins from different *Leishmania* species. For some regions of the proteins that had good homology (but not 100% identical), consensus sequences were obtained with CONS EMBOSS Explorer (http://www.bioinformatics.nl/cgi-bin/emboss/cons) and created according to the proximity of amino acids given by Taylor's classification [53]. Protein BLAST server (https://blast.ncbi.nlm.nih.gov/Blast.cgi) was used to analyze sequence similarity between *Leishmania* and human proteins in order to exclude protein regions with significant homology to human proteins.

### HLA-restricted T cell epitope prediction and design of multi-epitope peptides

To identify HLA-binding epitopes derived from each conserved or consensus protein sequence, NetMHC 3.2 server (http://www.cbs.dtu.dk/services/NetMHC-3.2/), NetMHCII 2.2 Server (http://www.cbs.dtu.dk/services/NetMHCII-2.2/) and IEDB T Cell Epitope Prediction Tools (http://tools.iedb.org/mhci/ and http://tools.iedb.org/mhcii/) were used. For HLA-I-binding epitopes, we have focused on 8, 9 or 10-mer long peptides. Each epitope was predicted for its binding affinity for 57 different HLA molecules (29 HLA-A and 28 HLA-B) considered to be the most frequent in the general worldwide population [54]. These molecules are representative of HLA allele sets, named supertypes, with common repertoires of peptide-binding motifs [55]. The IC50 values to determine the binding affinity of epitopes were calculated by utilizing IEDB and NetMHC 3.2 servers. A threshold of less than 50 nM was used to define strong binders (SB) and less than 500 nM to define weak binders (WB). HLA-I-restricted epitopes derived from each protein were arranged in a string of beads fashion, to construct multi-epitope peptides [56]. To optimize proteasome-mediated processing of these multi-epitope peptides, the proteasomal cleavage sites were predicted by the NetCHOP 3.1 (http://www.cbs.dtu.dk/services/NetChop/) and PAProC II (http://www.paproc2.de/paproc1/paproc2.html) algorithms. Linkers, also known as spacers, composed of some amino acids (ARY, RY, GR or TV), were added to improve peptide proteasomal cleavage for HLA-I presentation, if necessary [57, 58].

For HLA-II-binding epitopes, 15-mer long epitopes were predicted from each conserved or consensus protein sequence. Each epitope was predicted for its binding affinity for the most common HLA-II alleles classified in supertypes main DR (DRB1*0101, DRB1*0701, DRB1*0901, DRB1*1101, DRB1*1201, DRB1*1501, DRB5*0101 and DPB1*1401), DR4 (DRB1*0401, DRB1*0405 and DRB1*0802, DRB3 (DRB1*0301, DRB1*0301, DRB1*0301, 1302, DRB3*0101, DRB3*0202 and DRB4*0101), main DP (DPB1*0101, DPB1*0402 and DPB1*0501), and DP2 (DPB1*0201, and DPB1*0401) [59]. The IC50 values were calculated by utilizing IEDB and NetMHCII 2.2 servers. Hot spot areas of predicted HLA-II-restricted epitopes were selected within each conserved or consensus protein sequence to design long peptides. Global population coverage was estimated from a vaccine candidate composed with all designed multi-epitope peptides, by the IEDB Population coverage tools (http://tools.iedb.org/population/). Population coverage calculation was performed from 115 countries and 21 different ethnicities grouped into 16 different geographical areas.

### Physico-chemical properties and synthesis of Multi-epitope peptides

ExPASy ProtParam tool (https://web.expasy.org/protparam/) and Peptide property calculator (https://www.pepcalc.com/) were used to evaluate the physicochemical properties of the designed peptides such as hydrophobicity. The molecular weight of multi-epitope peptides was obtained by mass-spectrometry (Proteogenix, Schiltigheim, France). An ε-amino palmitoylation of a lysine (K) residue was introduced in N-terminal position of each designed multi-epitope peptide. These lipid tails were previously described to favor peptide presentation by antigen presenting cells (APC) [60]. Furthermore, it was reported that lipopeptides could constitute potent immunoadjuvants [61, 62]. In some designed multi-epitope peptides, the cysteines (S) of the original peptide sequence have been replaced by a glycine (G) or a serine (S) (underlined in the sequences) in order to avoid disulfide bridge formation, folding or multimerization of the peptide. To improve water-solubility, a hydrophobic spacer, as GR, was inserted between N-terminal lysine K and first epitope peptide [63].

The selected peptides were synthesized with more than 95% purity by Proteogenix (Schiltigheim, France).

Lyophilized peptides were dissolved according to their solubility in bi-distilled water, dimethyl sulfoxide (DMSO) or sodium bicarbonate, diluted in sterile double distilled water and stored at -80°C.

### Preparation of soluble *Leishmania* antigen (SLA)

SLA was prepared from cultures of stationary phase *L*. *major* promastigotes (MHOM/TN/94/GLC94). Parasites were washed in 1× phosphate-buffered saline (PBS), centrifuged at 1000× g/10 min at 4°C and supernatants were removed. The pellets were resuspended in lysis buffer (50 mM Tris/5 mM EDTA/HCl, pH7.1 ml/ 1 × 10^9^ parasites), subjected to three rapid freeze/thaw cycles followed by three pulses of 20 s/40 W with sonicator. Samples were centrifuged at 5000× g for 20 min at 4°C, and supernatants were collected, aliquoted and stored at -80°C until use. Protein quantification was performed using Bradford method.

### Cell culture and stimulation

Peripheral blood mononuclear cells (PBMCs) were isolated from heparinized blood by density centrifugation through Ficoll-Hypaque (GE Healthcare Bio-Sciences AB, Uppsala, Sweden). Cells were cultured at a concentration of 10^6^/ml in RPMI 1640 medium supplemented with 100 IU/ml penicillin, 100 μg/ml streptomycin, 2 mM L-glutamine and 10% heat inactivated human AB serum (Sigma-Aldrich, St Louis, MO). Cells were plated in 96 well culture plates (Greiner Bio-One, Germany) and incubated overnight in a 5% CO_2_ humidified atmosphere at 37°C for rest. Cells were then kept with media alone (unstimulated) or stimulated with either 1 μM of individual peptides or with peptide pools at 1μM/peptide. Recombinant human IL-2 (BD Bioscience, San Diego, CA) at 0.1 ng/ml final concentration was added after 24 hours and every 3 days along the culture period. Cell cultures were incubated for 10 days. As positive control cultures, cells were stimulated with Phytohemagglutinin (PHA) (Sigma-Aldrich, St Louis, MO) (10 μg/ml) and SLA (as indicator of previous disease) (10 μg/ml), for 5 days. Culture supernatants (in triplicate for each condition) were harvested and frozen at -80°C until use for ELISA and CBA assays, whereas cells were used for ELISPOT test and cell phenotyping.

### Enzyme Linked Immunosorbent Assay (ELISA)

IFN-γ and IL-10 levels were measured in culture supernatants of PBMC exposed for 10 days to individual peptides or peptide pools or for 5 days to PHA or SLA. Human IFN-γ or IL-10 ELISA Sets (BD Biosciences, San Diego, CA) were used according to the manufacturer’s instructions. Results were interpolated from a standard curve using recombinant cytokines and were expressed in pg/ml.

### Cytometric Bead Array assay (CBA)

TNF-α and GrB were quantified in culture supernatants of PBMC exposed for 10 days to peptide pools or 5 days to PHA or SLA, using the BD CBA Human Soluble Protein Flex Set system (BD Biosciences, San Diego, CA), according to the instructions of the manufacturer. In order to quantitate samples, the BD CBA Human Soluble Protein Flex Standard was performed for each cytokine and in each experiment. Data was acquired by flow cytometry (FACS Canto II, BD Bioscience, San Jose, CA). Flow Cytometric Analysis Program Array (FCAP Array; BD Biosciences) software was used for samples analysis.

### Intracellular cytokine staining and flow cytometry

After 10 days of *in vitro* stimulation with peptide pools, cells were washed and re-stimulated overnight with peptide pools (1 μM/peptide) and anti-CD49d/anti-CD28 antibodies (BD biosciences) as co-stimulators at 1 μg/ml final concentration. As positive control cultures, cells were stimulated with Phorbol Myristate Acetate (PMA) (50 ng/mL)/Ionomicyn (10^−6^ M) for 6 hours or with SLA (10 μg/ml) for 5 days. Cytokine secretion was stopped by Golgi stop (BD Biosciences, San Diego, CA) for the last 6 hours of culture. Cells were washed and incubated with anti-CD3-APC-H7 (SK7), anti-CD4-FITC (RPA-T4), anti-CD8-PE-Cy7 (RPA-T8), anti-CD45RO-APC (UCHL1) and anti-CCR7-PE (3D12) or with anti-CD3-APC-H7 (SK7), anti-CD4-FITC (RPA-T4), anti-CD8-PerCP-Cy5.5 (RPAT-8) or with anti-CD3-APC-H7 (SK7), anti-CD4-PerCP-Cy5.5 (RPA-T4) antibodies (BD biosciences, San Diego, CA), for 30 min at 4°C.

For intracellular staining, cells were washed, fixed and permeabilized using BD cytofix/Cytoperm plus kit (BD Biosciences, San Diego, CA) according to the manufacturer’s instructions and co-stained with anti-IFN-γ-Alexa Fluor 647 (B27) and anti-GrB-PE (GB11) or with anti-IFN-γ-Alexa Fluor 647 (B27), anti-TNF-α-Alexa Fluor 488 (MAb11) and anti-IL-2-PE (MQ1-17H12) antibodies (BD biosciences, San Diego, CA), for 30 min at 4°C. Data acquisition was performed on a FACS canto II flow cytometer (BD Bioscience, San Jose, CA) and analysis was performed with FlowJo 7.6 software (TreeStar, Ashland OR). For acquisition, CompBeads Set Anti-Mouse Ig, k (Anti-Mouse Ig, k/Negative Control (FBS) Compensation Particles Set) (BD Biosciences, San Diego, CA), were used.

### Enzyme Linked Immunospot Assay (ELISpot)

ELISpot assay was performed with PBMC that were stimulated for 10 days with peptides pools (1μM/peptide), or for 5 days with SLA (10 μg/ml) or PHA (10 μg/ml) (positive controls), using the human interferon-γ ImmunoSpot kit (CTL, Germany), according to the manufacturer’s recommendations. Briefly, 96-well PVDF filter plates were coated with human IFN-γ capture antibody. PBMCs (5.10^5^/ml) were washed, resuspended in CTL Test Medium and re-stimulated with peptide pools (1 μM/peptide) or with SLA (10 μg/ml) in the ELISPOT plates. PHA (10 μg/ml) and cells incubated with medium alone were used as positive and negative controls, respectively. Triplicate cultures were used. Cells were incubated for 24 hours in a 5% CO_2_ humidified atmosphere at 37°C. Biotinylated anti-IFN-γ detection antibody was added and plates were incubated for 2 hours at room temperature. After washing, streptavidin-alkaline phosphatase conjugate was added for 30 min at room temperature. The spots were revealed by adding the substrate of the enzyme. Spots were counted using CTL ImmunoSpot reader (CTL Analyzers, ShakerHights, OH). Results were expressed as spot forming units (SFU) per 10^6^ PBMC.

### Statistical analysis

Statistical analyses were performed with GraphPad Prism 5.0 (GraphPad Software, San Diego, CA). Results were expressed as medians (interquartile range, IQR as variances). Wilcoxon signed-rank test was used to compare median levels of cytokines between stimulated and unstimulated cultures or between percentages of cells producing cytokines after different stimulations. Mann-Whitney test was used for inter-groups analysis on normalized data after deducting the unstimulated value. p-values less than 0.05 were considered significant. Correlations were estimated using the Spearman rank (rs) correlation coefficient.

## Results

### *In silico* prediction of HLA-I restricted epitopes and design of multi-epitope peptides

We selected H2B, LmlRAB and PSA proteins as candidate antigens to map potential HLA-I restricted epitopes. In order to screen for conserved HLA-I restricted epitopes among *Leishmania* species, we first performed a protein sequence alignment from H2B and LmlRAB of *L*. *major* with corresponding protein sequences from *L*. *donovani*, *L*. *infantum*, *L*. *mexicana and L*. *braziliensis* species. The alignments showed 80.7 to 98.2% of identity for H2B and 63.6 to 90.8% of identity for LmlRAB. Protein sequence alignments from full *L*. *amazonensis* PSA sequence and PSA family from *L*. *infantum*, *L*. *donovani*, *L*. *tropica*, *L*. *chagasi* and *L*. *braziliensis*, showed 47.4 to 80.1% of identity. As we previously described, the central (LRR domain) and C-terminal regions of the PSA proteins are more divergent than the N-terminal region [64]. Predicted HLA-I restricted epitopes were selected from highly conserved regions of each protein or consensus sequence, according to (i) high binding scores from IEDB and NetMHC 3.2 servers (ii) promiscuity (epitopes presented by multiple HLA molecules from a supertype and, when it was possible, presented by multiple HLA-I supertypes), and (iii) low or no significant homology with human proteins. From these, a total of 17 epitopes were selected, among them 4, 6 and 7 HLA-I restricted epitopes were identified from H2B, LmlRAB and PSA, respectively (**Table 3**).

**Table 3:** Characteristics of *in silico* predicted HLA-I restricted epitopes derived from H2B, PSA and LmlRAB proteins.

Protein	Main epitope sequence	HLA-I supertype SB*(IC50)^a^	HLA-I supertype WB**(IC50)
**H2B**	**KAINAQMSM**	HLA-B27 (42), HLA-B58 (16)	HLA-A01/03 (107), HLA-B62 (293)
	**MERICTEAA**	HLA-B44 (24)	
	**MSMSHRTMK**	HLA-A01/A03 (41), HLA-A03 (5)	HLA-B27 (415)
	**SMSHRTMKI**	HLA-A02 (10)	HLA-A01 (110), HLA-B08 (165), HLA-B27 (235)
**PSA**	**TPEQRTNTL**	HLA-B07 (18), HLA-B27 (50)	HLA-B08 (68)
	**ELGKKWIG**	HLA-B08 (14)	
	**TLPEMPVGV**	HLA-A02 (2)	
	**PEMPAGVDY**		HLA-B44 (193)
	**ARGREGYFLA**		HLA-B27 (233)
	**EGYFVTDEK**	HLA-A03 (19)	
	**GARGREGY**		HLA-B58 (80)
**LmlRAB**	**RFAQGEHDI**		HLA-A24 (151)
	**RLPENAFVI**	HLA-A01 (26), HLA-A02 (4)	
	**TQGSSKAGF**		HLA-B27 (120), HLA-B62 (73)
	**MPHVDQSSI**	HLA-B07 (23)	
	**ASFRSTEAI**		HLA-A01 (103), HLA-B27 (62)
	**SSIMVVANK**	HLA-A03 (7)	HLA-A01/A03 (94)

^a^IC50 values (nM) were predicted by NetMHC 3.2 and IEDB

*Strong binders (IC50<50nM).

**Weak binders (50nM>IC50<500nM)

All the selected epitopes were predicted to have a strong or weak binding affinity for at least one of the HLA-I supertypes analyzed. To increase the coverage of the target population, we designed multi-epitopes peptides composed of HLA-I epitopes derived from each protein. We designed (i) one multi-epitope peptide containing four 9-mer HLA-I restricted epitopes derived from H2B (H2BI, 40-mer) (ii) two multi-epitope peptides, each containing three 9-mer HLA-I restricted epitopes derived from LmlRAB (RABI.2, 34-mer; RABI.3, 36-mer) and (iii) three multi-epitope peptides containing 8-, 9- or 10-mer HLA-I restricted epitopes derived from PSA (PSAI.3, 19-mer, 2 epitopes; PSAI.4, 18-mer, 2 epitopes and PSAI.5, 28-mer, 3 epitopes) (Patents WO/2014/102471, PCT/EP2019/064088) (**Table 4**).

**Table 4:** Characteristics of *in silico* predicted multi-epitope peptides.

Protein	Peptide name	Synthetic multi-epitope sequence	AA* number	MW** (g/mol)	Peptide-binding HLA-I supertype concatenation
**H2B**	**H2BI**	**KARY**MSMSHRTMKSMSHRTMKIKAINAQMSMMERICTEAA	40	4917.00	HLA-A01, HLA-A02, HLA-A01/A03, HLA-A03, HLA-B08, HLA-B27, HLA-B44, HLA-B58, HLA-B62
**PSA**	**PSAI.3**	**K**TPEQRTNTL**TV**ELGKKWIG	19	2538.10	HLA-B07, HLA-B08, HLA-B27
	**PSAI.4**	**K**TLPEMPVGVPEMPAGVDY	18	2268.83	HLA-A01, HLA-A02, HLA-A01/A24, HLA-B08, HLA-B44, HLA-B62
	**PSAI.5**	**K**ARGREGYFLARGARGREGYEGYFVTDEK	28	3579.13	HLA-A01, HLA-A02, HLA-A01/A03, HLA-A03, HLA-B27, HLA-B44, HLA-B58, HLA-B62
**LmlRAB**	**RABI.2**	**KARY**RFAQGEHDIRLPENAFVI**ARY**TQGSSKAGF	34	4123.82	HLA-A01, HLA-A02, HLA-A24, HLA-B27, HLA-B62
	**RABI.3**	**KARY**MPHVDQSSI**ARY**ASFRSTEAI**RY**SSIMVVANK	36	4373.20	HLA-A01, HLA-A01/A03, HLA-A03, HLA-B07, HLA-B27

*AA: Amino acid

**MW: Molecular weight (g/mol) obtained by mass-spectrometry

Letters in bold (K, KARY, ARY, RY, TV) represent spacers added to the multi-epitope sequence.

Underlined glycine (G) replaces a cysteine C

Additional amino acids (K, KGR, KARY, ARY, RY or TV) were introduced to improve peptide proteasomal cleavage or water-solubility, or to introduce lipid tail in N-terminal position.

In summary, we have designed 6 HLA-I-restricted multi-epitope peptides, which collectively were predicted to bind with strong or weak affinity to all HLA-I supertypes, providing large population coverage (95.55% estimated population coverage) (**Table 5**).

**Table 5:** Estimated population coverage.

Population/Area	HLA-I			HLA-II			Combined HLA-I/-II		
	Coverage^a^	Average_hit^b^	pc90^c^	Coverage^a^	Average_hit^b^	pc90^c^	Coverage^a^	Average_hit^b^	pc90^c^
World	95.55%	4.51	1.81	99.59%	10.39	5.6	99.98%	14.91	9.2

^a^Projected population coverage

^b^Average number of epitope hits / HLA combinations recognized by the population

^c^Minimum number of epitope hits / HLA combinations recognized by 90% of the population

### *In silico* prediction of hot spot regions rich in HLA-II restricted epitopes and design of HLA-II-restricted multi-epitope peptides

Hot spots of promiscuous 15-mer HLA-II restricted epitopes were mapped from highly conserved regions or consensus sequences of H2B, LmlRAB and PSA proteins. Selected HLA-II restricted peptides were compared against *L*. *amazonensis*, *L*. *infantum*, *L*. *donovani*, *L*. *tropica*, *L*. *braziliensis*, *L*. *mexicana*, *L*. *chagasi* and *L*. *major* (sequence homologies ranging from 40.74 to 100%). Peptides were further selected based on low or no significant homology with human proteins.

One multi-epitope peptide derived from H2B (H2BII, 34-mer), two multi-epitope peptides derived from LmlRAB (RABII.4, 24-mer, RABII.5, 35-mer) and two multi-epitope peptides derived from PSA (PSAII.6, 29-mer, PSAII.7, 31-mer) (Patent PCT/EP2019/064088), were designed (**Table 6**).

**Table 6:** Characteristics of *in silico* predicted sequence rich in HLA-II–restricted epitopes derived from H2B, PSA and LmlRAB proteins.

Protein	Peptide name	Sequence rich in HLA-II epitopes	AA number	MW (g/mol)	HLA-II supertype SB*(IC50)^a^	HLA-II supertype WB**(IC50)
**H2B**	**H2BII**	KGRKPKRSWNVYVGRSLKAINAQMSMSHRTMKIV	34	4199.23	DR1: DRB1*0101 (4), DR7: DRB10701 (19), DR9: DRB1*0901 (43), DR11/DR12: DRB1*1101/DRB1*1201 (44), DRB5: DRB5*0101 (43), DRB4: DRB4*0101 (13)	DR15:DRB1*1501 (62), DR4: DRB1*0401 (86), DR4: DRB1*0405 (87), DR4: DRB1*0802 (127), DR3: DRB1*0301 (64), DR13: DRB1*1302 (300), DP1: DPB1*0101 (313), DP402: DPB1*402 (415)
**PSA**	**PSAII.6**	KGRAARSARSGEGYFLTDEKTSLVYGDGG	29	3287.76	DR3: DRB1*0301 (8), DRB3: DRB3*0101/DRB3*0202 (26)	DR1: DRB1*0101 (123), DR7: DRB10701 (342), DR11/DR12: DRB1*1101/DRB1*1201 (156), DR4: DRB1*0401 (70), DP1: DPB1*0101 (302), DP402: DPB1*402 (382)
	**PSAII.7**	KGREMPSGSDYSHSMIRDLDFSNMGLLLSGT	31	3641.28	DR1: DRB1*0101 (39), DR7: DRB10701 (14), DR4: DRB1*0401 (22)	DR9: DRB1*0901 (65), DR11/DR12: DRB1*1101/DRB1*1201 (275), DR15: DRB1*1501 (329), DRB5: DRB5*0101 (215), DR4: DRB1*0405 (64), DR4: DRB1*0802 (317), DR3: DRB1*0301(263), DR13: DRB1*1302 (197), DRB4 (146), DRB3: DRB3*0101/DRB3*0202 (130), DP1: DPB1*0101 (98), DP402: DPB1*402 (30), DP2: DPB1*0201 (374), DP401: DPB*0401 (224)
**LmlRAB**	**RABII.4**	KGRLHSVVGRHLSIVADHMPHLDQ	24	2941.58	DR1: DRB1*0101 (13), DR7: DRB10701 (6), DR13: DRB1*1302 (27), DRB3: DRB3*0101/DRB3*0202 (16), DRB4: DRB4*0101 (48)	DR9: DRB1*0901 (424), DR11/DR12: DRB1*1101/DRB1*1201 (86), DR15: DRB1*1501 (74), DRB5: DRB5*0101 (177), DR4: DRB1*0401 (67), DR4: DRB1*0405 (196), DR4:DRB1*0802 (292), DR3: DRB1*0301 (133)
	**RABII.5**	KGRSENAIVTSRKVQEEVFDLFTDVHYSEVSAKTK	35	4237.91	DR7: DRB*10701 (31), DR11/DR12: DRB1*1101/DRB1*1201 (32), DR3: DRB1*0301 (38), DP1: DPB1*0101 (34), DP2: DPB1*0201 (11), DP401: DPB*0101(32)	DR1: DRB1*0901 (286), DR9: DRB1*0901 (400), DR15: DRB1*1501 (141), DRB5: DRB5*0101 (59), DR4: DRB1*0401 (63), DR4:DRB1*0405 (272), DR4:DRB1*0802 (199), DRB3: DRB3*0101/DRB3*0202 (274) DP402: DPB1*402 (259), DP5: BPB1*0501 (84)

^a^ IC50 values (nM) were predicted by NetMHCII 2.2 and IEDB.

*Strong binders.

**Weak binders.Underlined serine (S) replaces a cysteine C.

Additional amino acids (K or KGR) were introduced to improve peptide water-solubility or to introduce lipid tail in N-terminal position. Peptides rich in HLA-II epitopes were predicted to bind with strong or weak affinity to the most frequent 18 HLA-II alleles, representative of HLA-II supertypes. In total, we have designed 5 HLA-II restricted multi-epitope peptides with strong or weak affinity to all HLA-II supertypes, providing wide population coverage estimated to 99.59% (**Table 5)**. It should be noted that the designed multi-epitope peptides, having a high affinity for several HLA-I and -II molecules, if taken all together as vaccine candidate components, could have a strong immunoprevalent potential in leishmaniasis endemic areas (**Table 5**).

### Cytokine responses to multi-epitope peptides in cured CL subjects

We evaluated the ability of the multi-epitope peptides, used either individually (N = 11) or as peptide pools (N = 25), to induce IFN-γ and IL-10, in individuals with cured CL and in healthy subjects, by ELISA.

Peptides pools have been constituted to group HLA-I, HLA-II or HLA-I and HLA-II peptides, derived from the same or different proteins (**Table 7**).

**Table 7:** Peptide pools composition.

Protein	H2B		PSA					RABGTPase			
Peptides	H2BI	H2BII	PSAI.3	PSAI.4	PSAI.5	PSAII.6	PSAII.7	RABI.2	RABI.3	RABII.4	RABII.5
**Pools including HLA-I peptides**											
PI			x	x	x						
RI								x	x		
P1	x		x	x	x						
P2	x							x	x		
P3			x	x	x			x	x		
P4	x		x	x	x			x	x		
**Pools including HLA-II peptides**											
PII						x	x				
RII										x	x
P5		x				x	x				
P6		x								x	x
P7						x	x			x	x
P8		x				x	x			x	x
**Pools including HLA-I and HLA-II peptides**											
P9	x	x									
P10			x	x	x	x	x				
P11								x	x	x	x
P12	x					x	x				
P13	x	x				x	x				
P14	x					x	x	x	x		
P15		x		x	x					x	x
P16	x	x	x	x	x	x	x				
P17	x	x						x	x	x	x
P18	x			x	x					x	x
P19		x				x	x	x	x		
P20			x	x	x	x	x	x	x	x	x
P21	x	x	x	x	x	x	x	x	x	x	x

**Fig 1:**
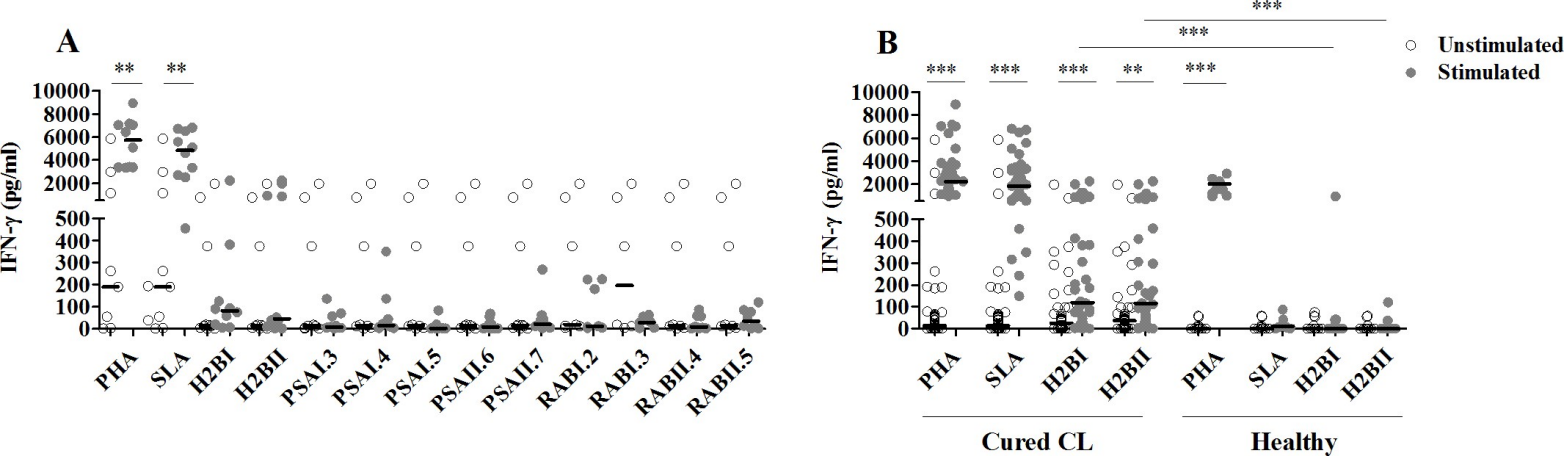
IFN-γ responses to multi-epitope peptides. IFN-γ was quantified by ELISA, in the culture supernatants of PBMC from cured CL subjects (N = 10) stimulated with multi-epitope peptides (1 μM), for 10 days (**A**). PHA and SLA (10 μg/ml) were used as positive controls. Unstimulated cultures (medium) were used as negative controls. H2BI and H2BII peptides were further evaluated in a larger number of cured CL (N = 40) as well as in healthy individuals (N = 12) (**B**). Horizontal bars represent median values. Statistical significance was assigned to a value of p<0,05 (**p<0.01, ***p<0.001).

As expected, high significant levels of IFN-γ were observed in cured CL, but not in healthy individuals, in response to SLA stimulation (p<0.0001) (**Fig 1A and 1B**).

No significant differences in IFN-γ induction were observed between stimulated and unstimulated cultures, after stimulation of PBMC from ten cured CL individuals with multi-epitope peptides used individually (**Fig 1A**). However, among these peptides, H2BI and H2BII showed the highest IFN-γ levels, although not statistically significant when compared to unstimulated cultures. We further analyzed these 2 peptides in a larger group of healed CL and in healthy individuals. As shown in Fig 1, significant levels of IFN-γ were detected in healed CL, when compared to unstimulated cultures (**Fig 1B**). In addition, H2BI and H2BII were not able to induce a significant IFN-γ response in healthy individuals (**Fig 1B**).

**Fig 2:**
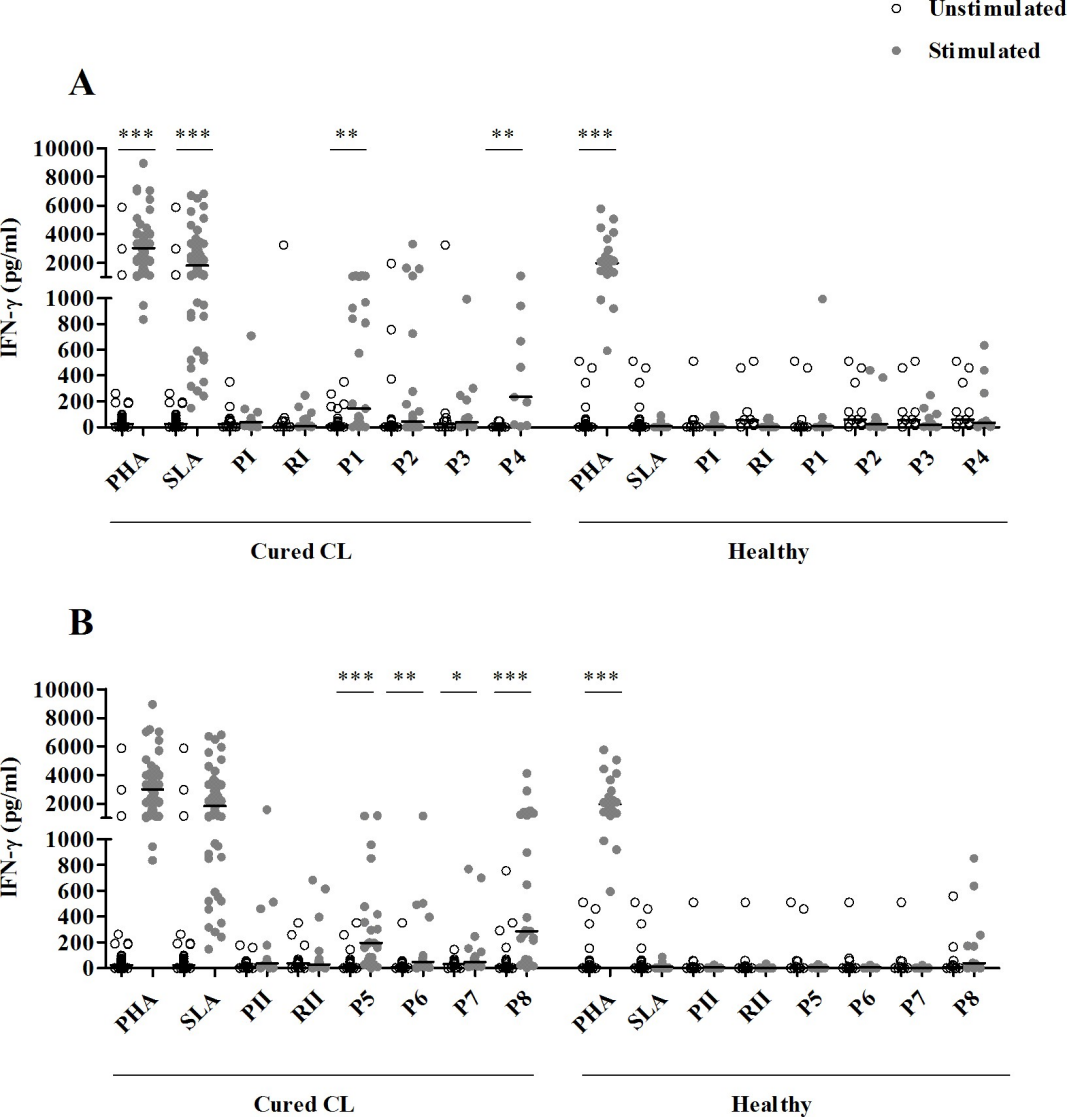
IFN-γ responses to pools composed of HLA-I or HLA-II peptides. IFN-γ was quantified by ELISA, in culture supernatants of PBMC from cured CL subjects (N = 23) or healthy individuals (N = 12) stimulated with peptide pools (1 μM/peptide) composed of HLA-I (**A**) or HLA-II (**B**) peptides, for 10 days. PHA and SLA were used as positive controls (10 μg/ml). Horizontal bars represent median values. Statistical significance was assigned to a value of p<0,05 (*p<0.05, **p<0.01, ***p<0.001).

**Fig 3:**
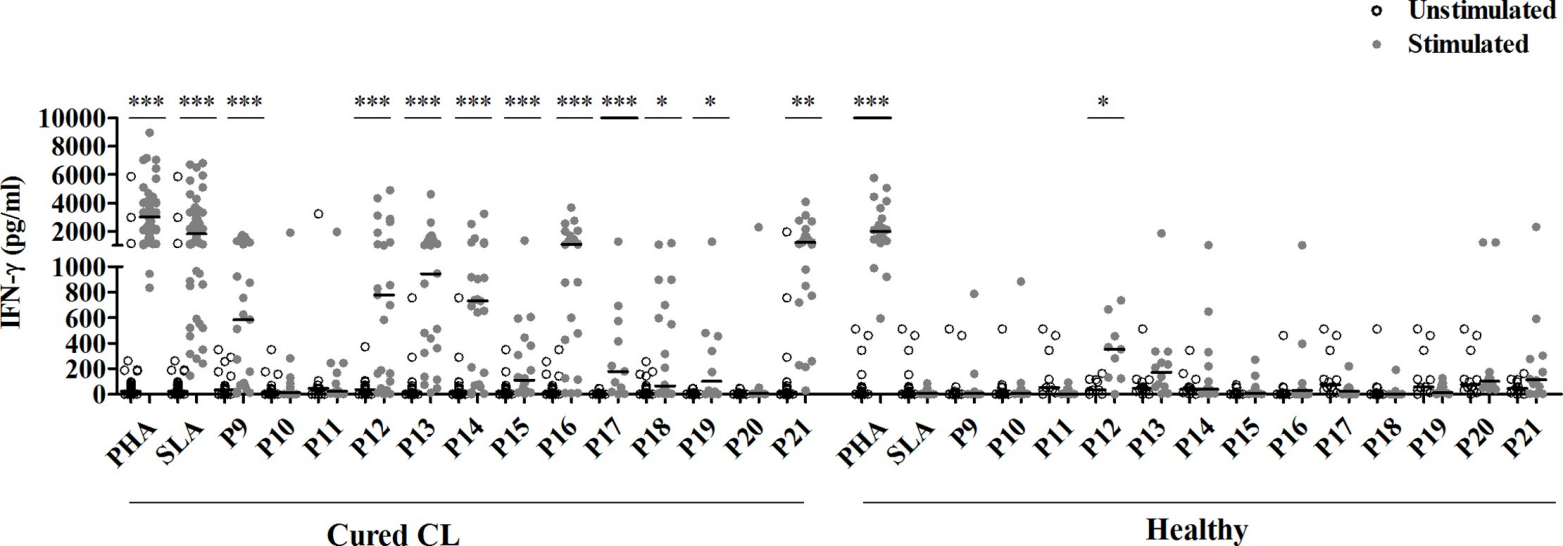
IFN-γ responses to pools composed of both HLA-I and HLA-II peptides. IFN-γ was quantified by ELISA, in culture supernatants of PBMC from cured CL subjects (N = 23) or healthy individuals (N = 12) stimulated with peptide pools (1 μM/peptide) composed of both HLA-I and HLA-II peptides, for 10 days. PHA and SLA were used as positive controls (10 μg/ml). Horizontal bars represent median values. Statistical significance was assigned to a value of p<0,05 (*p<0.05, **p<0.01, ***p<0.001).

Significant levels of IFN-γ were observed in response to two HLA-I (P1 and P4) (**Fig 2A**), four HLA-II (P5, P6, P7 and P8) (**Fig 2B**) and ten HLA-I/HLA-II peptide pools (P9, P12, P13, P14, P15, P16, P17, P18, P19 and P21) (**Fig 3**).

These responses were specific as no significant IFN-γ levels were detected in healthy individuals, except for P12 (**Figs 2 and 3**). This peptide pool induced significant levels of IFN-γ in cured CL as well as in the healthy group, suggesting that this response was not a feature of *Leishmania* infection. Interestingly, IFN-γ levels induced by P21 pool (including all multi-epitope peptides) in cured CL individuals (median/IQR: 1207/928) were similar to those induced by SLA in the same group (1182/1837), (p>0.05) (**Fig 3**).

**Fig 4:**
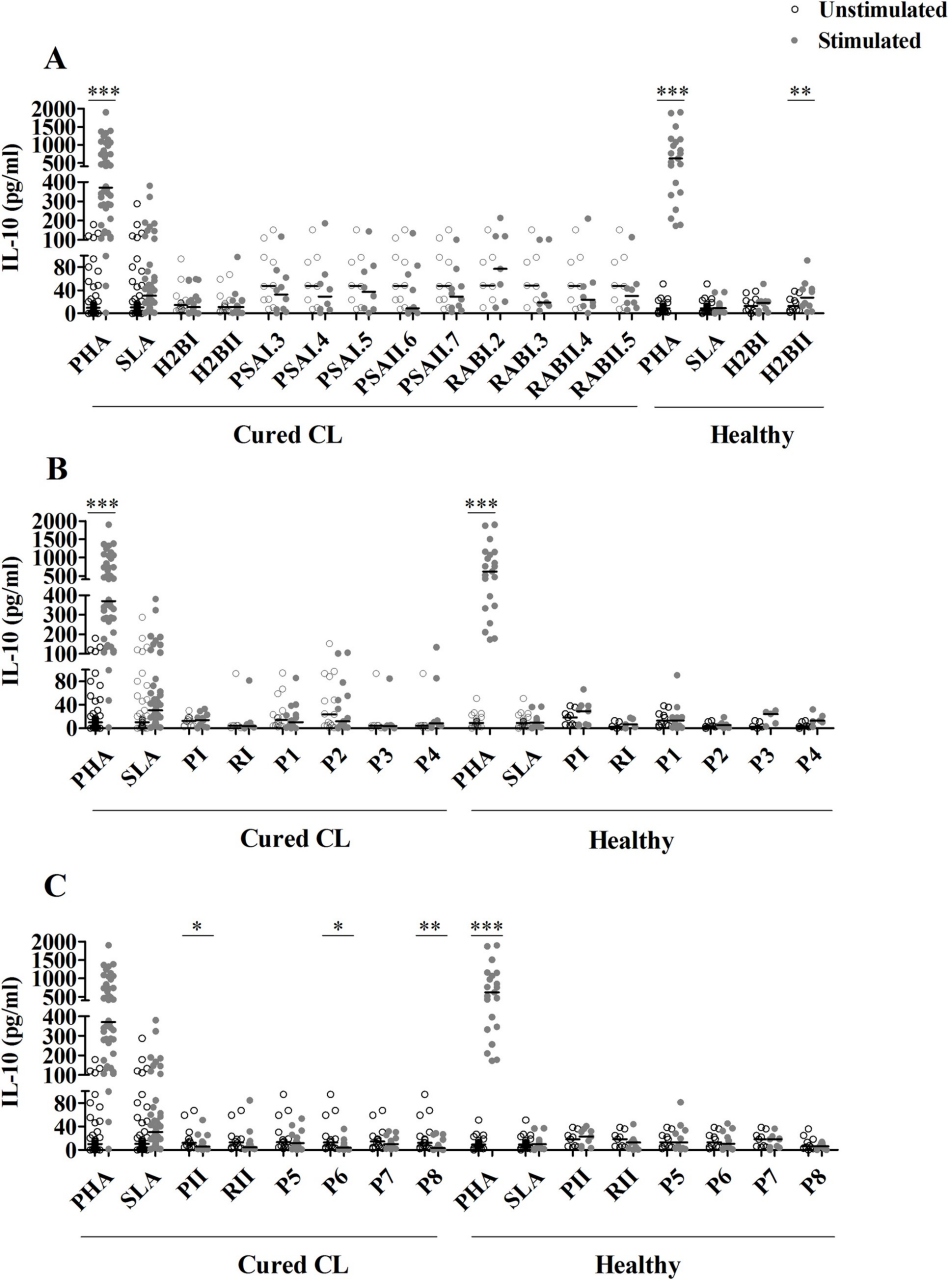
IL-10 responses to multi-epitope peptides used individually or as pools composed of HLA-I or HLA-II peptides. IL-10 was quantified by ELISA, in culture supernatants of PBMC from cured CL subjects (N = 16) or healthy individuals (N = 10) stimulated with multi-epitope peptides (1 μM) used individually **(A)**, or as peptide pools composed of HLA-I **(B)** or HLA-II **(C)** peptides, for 10 days. PHA and SLA were used as positive controls (10 μg/ml). Horizontal bars represent median values. Statistical significance was assigned to a value of p<0,05 (*p<0.05, **p<0.01, ***p<0.001).

**Fig 5:**
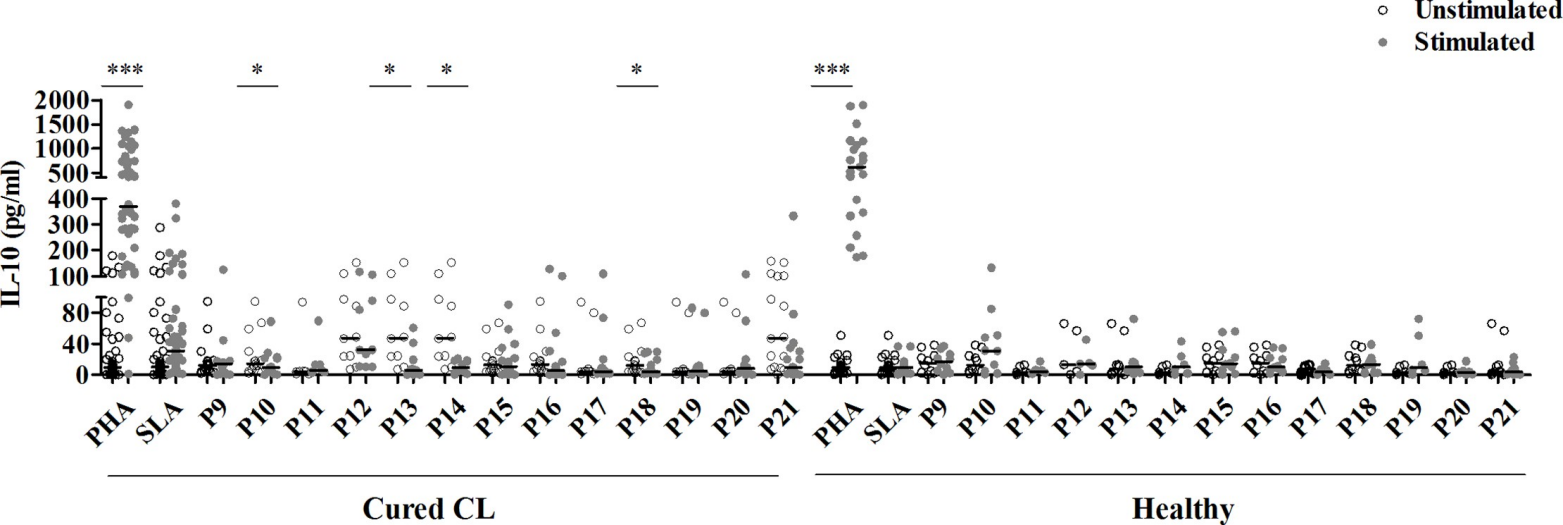
IL-10 responses to pools composed of both HLA-I and HLA-II peptides. IL-10 was quantified by ELISA, in culture supernatants of PBMC from cured CL subjects (N = 16) or healthy individuals (N = 10) stimulated with peptide pools (1μM/peptide) composed of both HLA-I and HLA-II peptides, for 10 days. PHA and SLA were used as positive controls (10 μg/ml). Horizontal bars represent median values. Statistical significance was assigned to a value of p<0,05 (*p<0.05, **p<0.01, ***p<0.001).

No IL-10 was observed in response to peptides used either individually (except low but significant levels observed for H2BII) or as peptide pools (**Figs 4 and 5**).

However, a suppressive effect on IL-10 production was observed after stimulation with PII, P6, P8, P10, P13, P14 and P18 (**Figs 4C and 5**).

**Fig 6:**
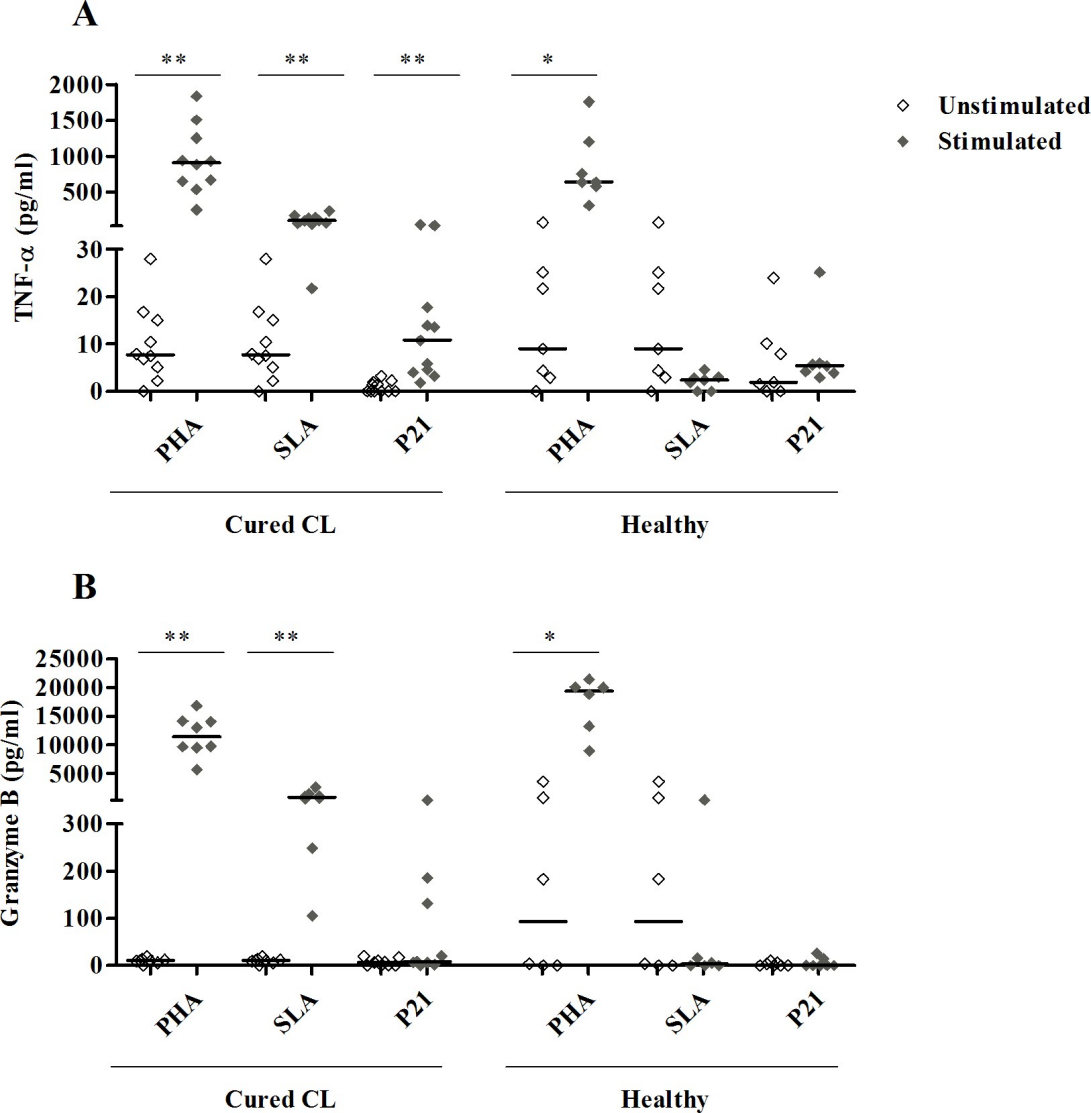
TNF-α and Granzyme B responses to P21 peptide pool. TNF-α **(A)** and GrB **(B)** were quantified by CBA, in the culture supernatants of PBMC from cured subjects (N = 11) or healthy individuals (N = 7) stimulated with P21 (1 μM), for 10 days. PHA and SLA (10 μg/ml) were used as positive controls. Horizontal bars indicate median values. Statistical significance was assigned to a value of p<0,05 (*p<0.05, **p<0.01).

We further analyzed TNF-α and GrB, by CBA, in response to P21 pool, in culture supernatants from PBMC issued from cured CL and healthy individuals (**Fig 6**).

Significant TNF-α levels were induced by P21 in cured (median/IQR in stimulated; unstimulated cultures: 10/13; 0/1.9) (p = 0.001), but not in healthy individuals (5.4/2; 1.9/10) (p>0.05) (**Fig 6A**). No GrB response was observed after P21 stimulation in cured CL individuals (**Fig 6B**). High and significant levels of TNF-α and GrB were observed in cured CL but not in healthy individuals, in response to SLA stimulation (p<0.005) (**Fig 6**).

For further analysis, and because PBMC numbers was a limiting factor, we selected peptide pools inducing the highest significant and specific IFN-γ levels. These pools included P21 (median/IQR in stimulated; unstimulated cultures: 1207/928; 15/93), P16 (1080/1249; 12/53), P13 (946/1229; 12/65), P14 (730/1052; 12/65), P9 (584/1148; 33/64), and P8 (287/1177; 15/64) (p <0.0003). Among these pools, P21, P16 and P13, sharing H2BI, H2BII and PII, induced the highest IFN-γ levels, suggesting that the combination of these peptides would be sufficient to induce IFN-γ levels as high as those induced by the P21 pool that contains 11 peptides.

### Quantification of specific IFN-γ producing T lymphocytes by ELISpot assay

**Fig 7:**
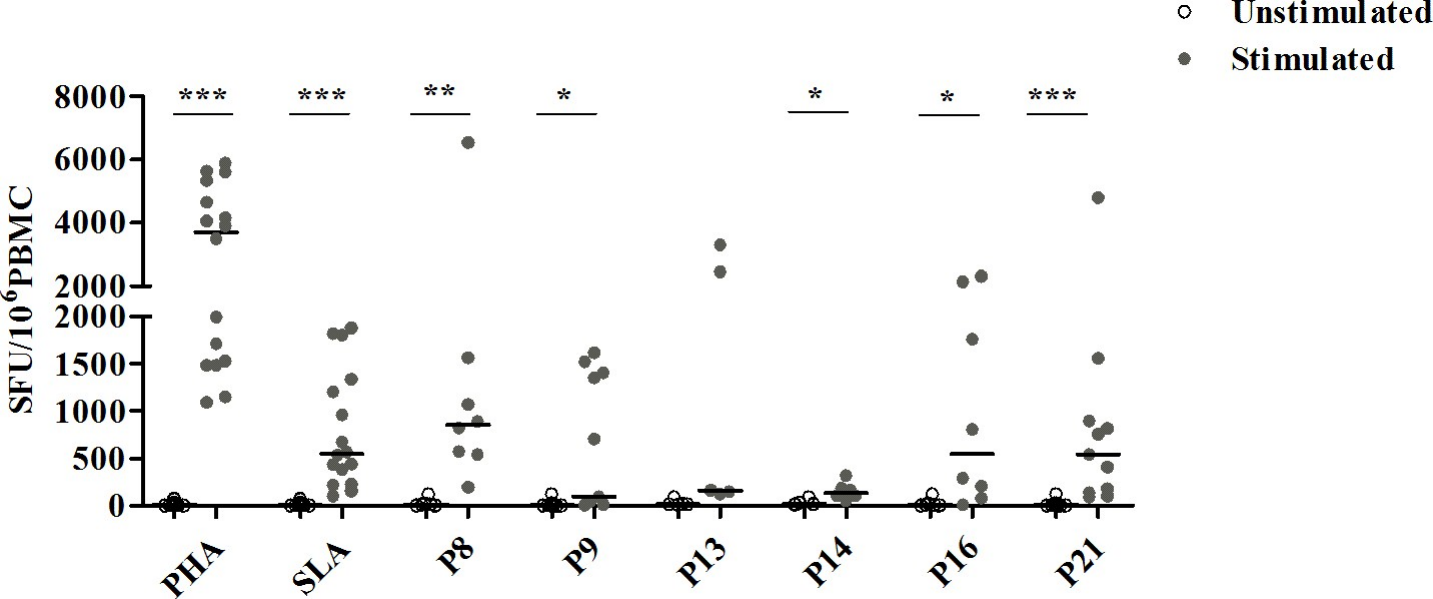
Enumeration of IFN-γ-secreting T cells in response to selected multi-epitope peptide pools. PBMC from cured CL subjects (N = 11), previously stimulated with selected peptide pools (1 μM/peptide) for 10 days, were re-stimulated for 24 h with the same peptide pools at the same concentration. PHA and SLA were used as positive controls (10 μg/ml). Results are expressed as spot forming units (SFU) per million PBMCs. Horizontal bars represent median values. Statistical significance was assigned to a value of p<0,05 (*p<0.05, **p<0.01, ***p<0.001).

The ability of multi-epitope peptide pools to activate *Leishmania*-reactive CD4+ T cells producing IFN-γ was assessed by ELISpot assay. Results were expressed as SFU per 10^6^ PBMC and compared between stimulated and unstimulated cultures. We showed significant responses against peptide pools P8 (median/IQR in stimulated; unstimulated cultures: 857/893; 8/24), P9 (95/1392; 10/27), P14 (135/125; 20/31), P16 (550/1933; 8/22) and P21 (542/762; 10/27) (p≤0.03) (**Fig 7**).

Interestingly, when compared to SLA induced response; no statistically significant difference was observed for P8, P9, P14, P16 and P21 pools. The IFN-γ response induced by P13 was not significantly increased when compared to unstimulated culture. This peptide pool could only be analyzed in 5 individuals and may need further analysis in a larger group of individuals to validate this negative result.

### HLA-typing and IFN-γ response

HLA typing was performed for five cured CL individuals. IFN-γ responses to P8, P9, P16 and P21 stimulation, were analyzed by ELISPOT (**S1 Table**). SFU/10^6^PBMC were considered positive if above the threshold defined as mean values observed in unstimulated cultures +2SD. A positive IFN-γ response was observed in response to P8, P9, P16 and P21 in four among five cured CL individuals (**S1 Table**). In contrast, no IFN-γ response was detected in CCL64 individual, after stimulation with P9 composed of H2BI and H2BII peptides. This donor does not express HLA-I alleles to which H2BI epitopes are able to bind, but carries HLA-II alleles for which H2BII epitopes have a strong affinity. The positive response observed to P8, P16 and P21, in this individual, could be induced by HLA-II-restricted epitopes derived from PSA protein shared by these peptide pools.

### Frequencies of specific cytokine secreting T cells after multi-epitope peptide stimulation

**Fig 8:**
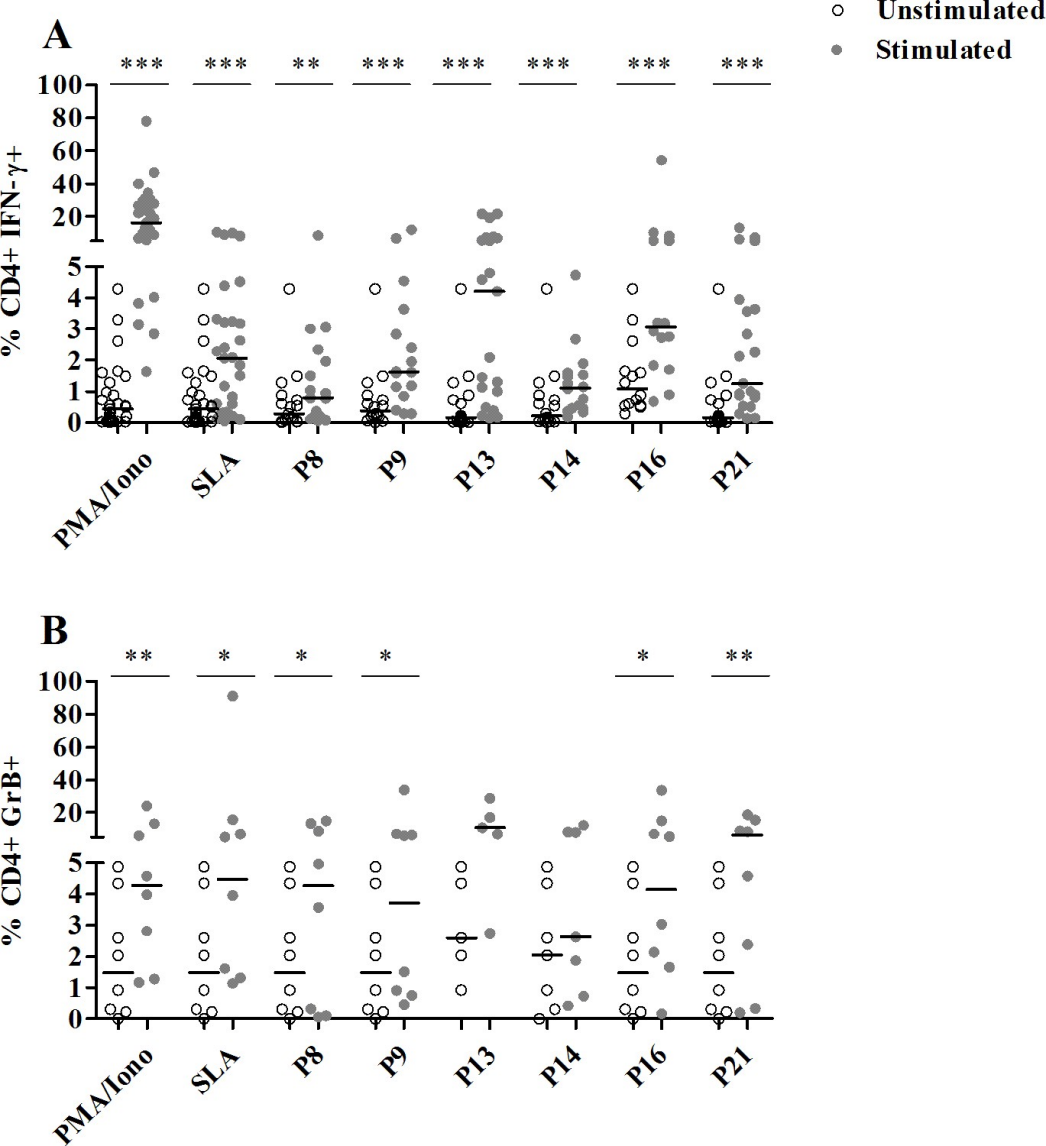
Percentages of CD4+ T cells producing IFN-γ or GrB in response to selected multi-epitope peptide pools. PBMC from cured CL subjects, previously stimulated with selected peptide pools (1 μM/peptide) for 10 days, were re-stimulated overnight with the same peptide pools (1 μM/peptide) and with anti-CD49d/anti-CD28 antibodies (1 μg/ml). For intracellular IFN-γ (N = 21 cured individuals) and Granzyme B (N = 9 cured individuals) detection, cells were treated with Golgistop for 6 h of culture, fixed and permeabilized using BD Cytoperm/cytofix kit. Data were analyzed by FlowJo software. PBMC stimulated with PMA (50 ng/ml)/Ionomicyn (10^–6^M) for 6 h or SLA (10μg/ml) for 5 days, were used as positive control. Results represent the frequency of CD4+ IFN-γ+ **(A)** and CD4+ granzyme B+ **(B)** T cell populations. Horizontal bars represent median values. Statistical significance was assigned to a value of p<0,05 (*p<0.05, **p<0.01, ***p<0.001).

PBMC stimulated by the selected peptide pools were stained and analyzed by flow cytometry to first determine the percentage of IFN-γ or GrB-producing CD4+ T cells among total CD4+ T cells, in cured CL subjects. We observed a significantly higher percentage of CD4+T cells producing IFN-γ in response to all selected peptide pools, in comparison to unstimulated cultures (median/IQR in stimulated; unstimulated cultures: P8: 0.7/1.9; 0.2/0.7, P9: 1.6/2.5; 0.3/0.6, P13: 4/6; 0.1/0.6, P14: 1.1/1.1; 0.2/0.7, P16: 3/4; 1/1.3 and P21: 1.2/3.1; 0.1/0.6) (p≤0.009) (**Fig 8A and S1 Fig**).

With regard to CD4+ T cells producing GrB, we showed a significant increase in their percentages after stimulation with P8 (4/12; 1.4/3.6), P9 (3.7/6.1; 1.4/3.6), P16 (4/11; 1.4/3.6) and P21 (6/12; 1.4/3.6) (p≤0.03) (**Fig 8B and S1 Fig**). The response induced by P13 peptide pool (10/18; 2/3) was not statistically significant. This result may need to be further studied in a larger group of individuals in order to be validated. We also assessed IFN-γ+/GrB+ CD4+ T cells among total CD4+ T cells and we showed a significant increase for this population after P21 stimulation (1.7/3.1; 0.1/0.5) (p = 0.01) (**S2 Fig**). No correlations were observed between IFN-γ+ CD4+ T cells and GrB+ CD4+ T cells.

**Fig 9:**
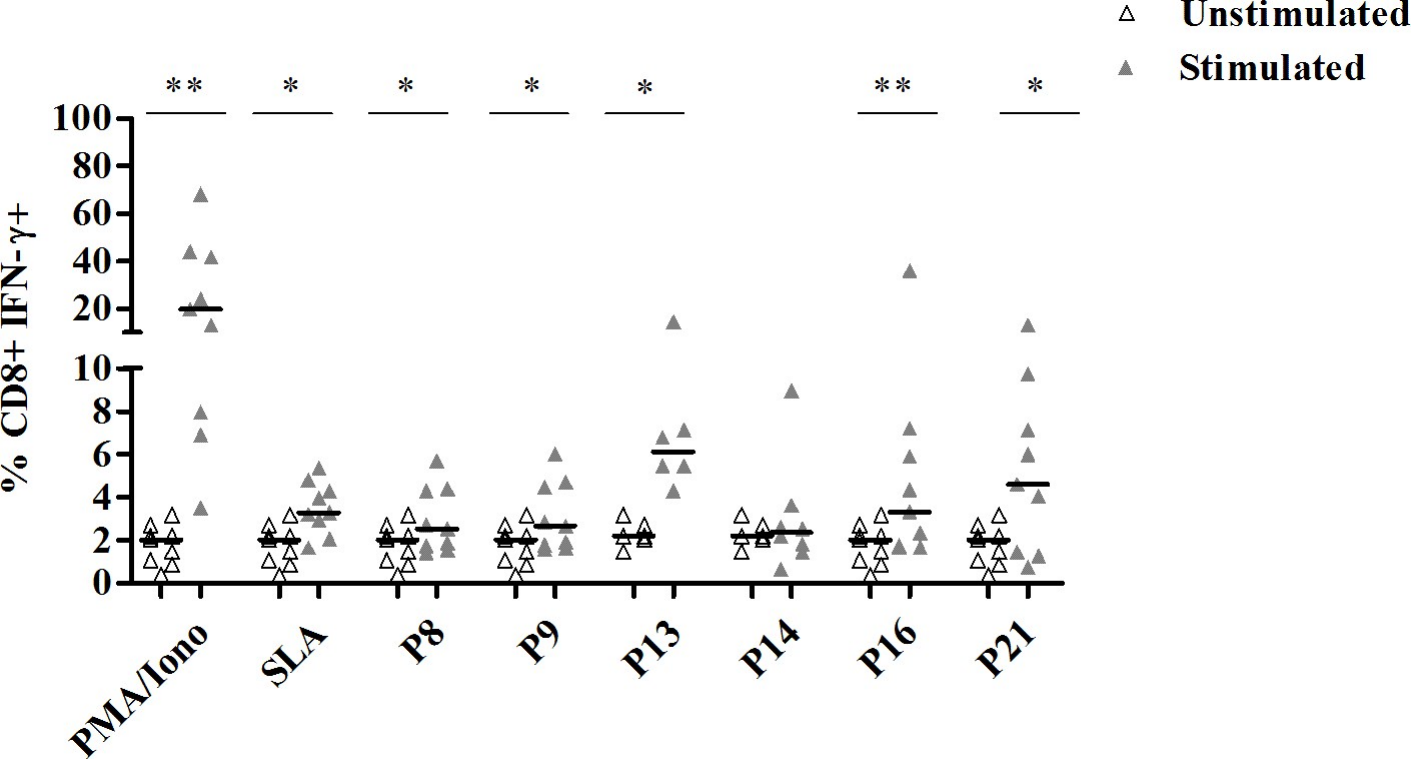
Percentages of CD8+ T cells producing IFN-γ in response to selected multi-epitope peptide pools. PBMC from cured CL subjects (N = 9), previously stimulated with selected peptide pools (1 μM/peptide) for 10 days, were re-stimulated overnight with the same peptide pools (1 μM/peptide) and with anti-CD49d/anti-CD28 antibodies (1 μg/ml). For intracellular IFN-γ detection, cells were treated with Golgistop for 6 h of culture, fixed and permeabilized using BD Cytoperm/cytofix kit. Data were analyzed by FlowJo software. PBMC stimulated with PMA (50 ng/ml)/Ionomicyn (10^–6^M) for 6 h or SLA (10 μg/ml) for 5 days, were used as positive control. Results represent the frequency of CD8+ IFN-γ+ T cell populations. Horizontal bars represent median values. Statistical significance was assigned to a value of p<0,05 (*p<0.05, **p<0.01).

We next assessed intracellular IFN-γ production by peptide-specific CD8+ T cells in cured CL individuals (**Fig 9 and S3 Fig**).

Results were expressed as percentages of IFN-γ+ CD8+ T cells among total CD8+ T cells and were compared between stimulated and unstimulated cultures. As shown in Fig 9, a significant increase in the percentage of IFN-γ+ CD8+ T cells was observed after stimulation with P8 (median/IQR in stimulated; unstimulated cultures: 2.5/2.6; 2/1.4), P9 (2.6/2.8; 2/1.4), P13 (6.1/3.8; 2.1/0.9), P16 (3.3/4.8; 2/1.4) and P21 (4.6/7; 2/1.4) (p≤0.03).

**Fig 10:**
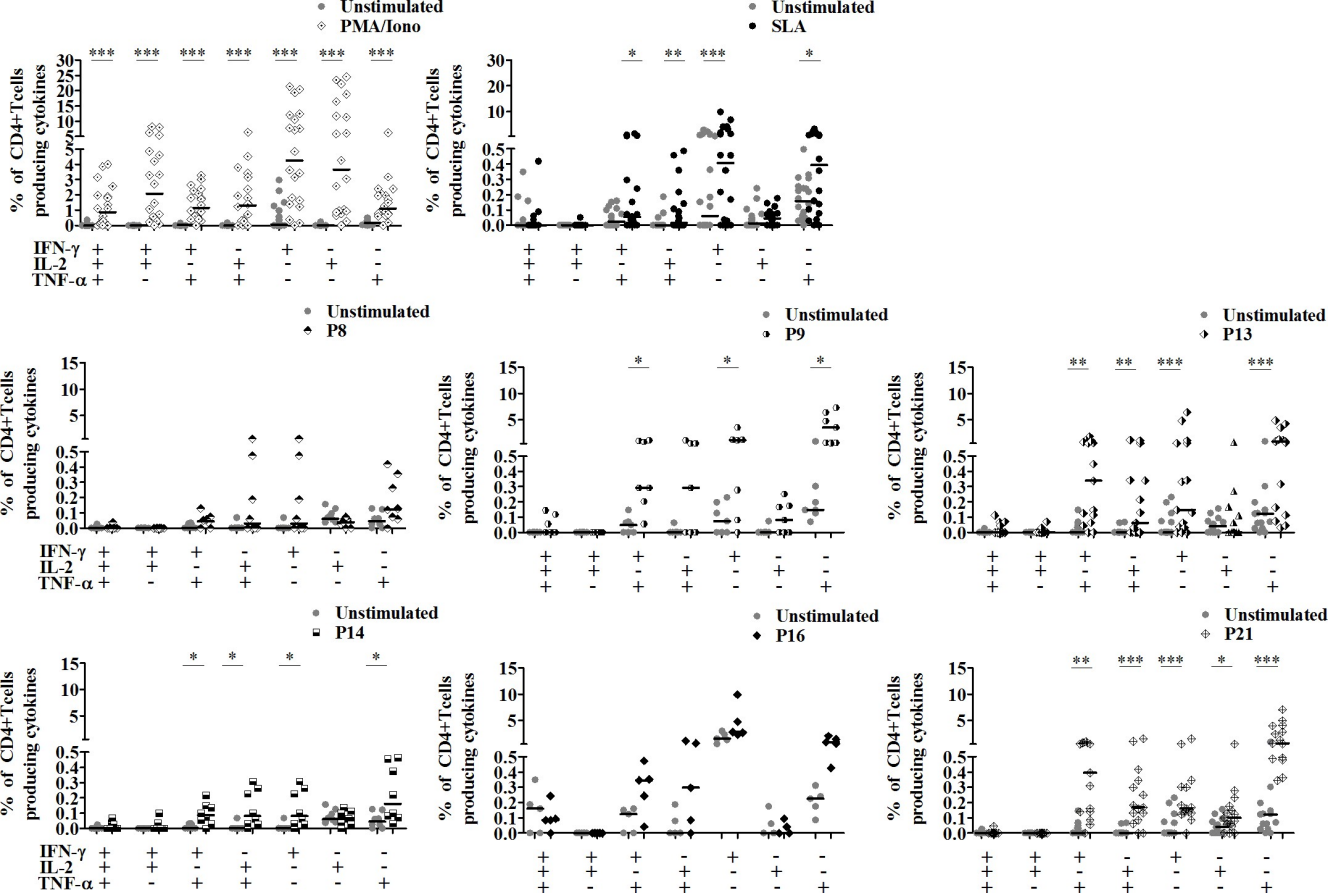
Multifunctional CD4+ T cell responses after multi-epitope peptide pools stimulation. PBMC from cured CL subjects (5 to 15 individuals), previously stimulated with selected peptide pools (1 μM/peptide) for 10 days were re-stimulated overnight with the same peptide pools (1 μM/peptide) and with anti-CD49d/anti-CD28 antibodies (1 μg/ml). For intracellular cytokine detection, cells were treated with Golgistop for 6 h of culture, fixed and permeabilized using BD Cytoperm/cytofix kit. Data were analyzed by FlowJo software. PBMC stimulated with PMA (50 ng/ml)/Ionomicyn (10^–6^M) for 6 h or SLA (10μg/ml) for 5 days, were used as positive control. A five-color flow cytometry panel was used to simultaneously analyze multiple cytokine at the single cell level. Boolean combinations of the three cytokine gates (IFN-γ, IL-2 and TNF-α) were used to uniquely discriminate responding cells based on their functionality. The frequency of CD4+ T cells expressing each of the seven possible combinations of IFN-γ, IL-2 and TNF-α was determined. Horizontal bars represent median values. Statistical significance was assigned to a value of p<0,05 (*p<0.05, **p<0.01, ***p<0.001).

Finally we evaluated the ability of peptide pools to stimulate a multifunctional CD4+ T cell response, using Boolean gating (**S4 Fig**). As shown in Fig 10, no significant increase was observed in the percentage of CD4+ T cells producing IFN-γ, IL-2 and TNF-α, in response to both peptide pools and SLA in cured CL individuals.

We also investigated whether these T cells display a monofunctional or bifunctional profile in response to peptide stimulation. A significant increase in the frequencies of bifunctional IFN-γ+/TNF-α+, IL-2+/TNF-α+, and monofunctional IFN-γ+ and TNF-α+ CD4+ T cells, was shown in response to SLA and to peptide pools P13, P14 and P21 (p<0.04) (**Fig 10**). P9 pool was also able to induce high significant percentages of IFN-γ+/TNF-α+, IFN-γ+ and TNF-α+ CD4+ T cells (**Fig 10**). Surprisingly, we did not detect any changes in these populations in response to P16 pool, probably due to the low number of individuals analyzed. P21 was the only pool to induce a significant increase in the IL-2+ CD4+ T cell population.

### Frequencies of specific memory T cells after multi-epitope peptide stimulation

**Fig 11:**
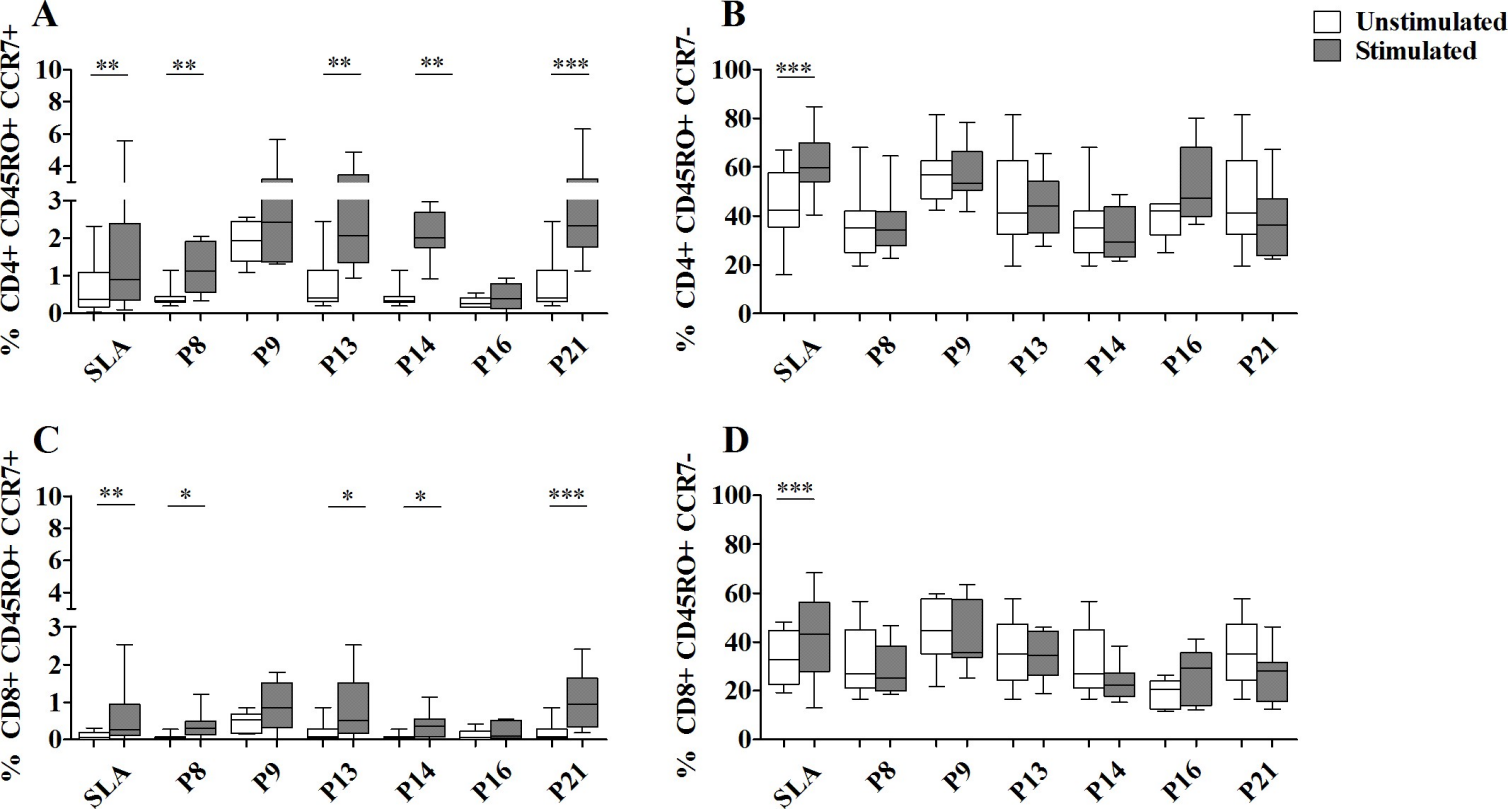
Phenotyping of multi-epitope peptide-specific CD4+ and CD8+ memory T cells. PBMC from cured CL subjects (N = 11), previously stimulated with selected peptide pools (1 μM/peptide) for 10 days, were re-stimulated overnight with the same peptide pools (1 μM/peptide) and with anti-CD49d/anti-CD28 antibodies (1 μg/ml). The percentages of CD45RO+CCR7+CD4+ or CD8+ TCM (**A, C**) and, CD45RO+CCR7-CD4+ or CD8+ TEM (**B, D**), were analyzed by FlowJo software. PBMC stimulated with SLA (10 μg/ml) for 5 days, were used as positive control. Statistical significance was assigned to a value of p<0,05 (*p<0.05, **p<0.01, ***p<0.001).

Phenotypic analysis of memory T cells in response to the selected multi-epitope peptide pools (N = 6) was performed in cured CL individuals (**Fig 11 and S5 Fig**).

We used CD45RO and CCR7 to distinguish T memory T cells into TCM (CD45RO+CCR7+) or TEM (CD45RO+CCR7-). Frequencies of peptide-specific TCM and TEM were assessed among total CD4+ or CD8+ T cell subsets. A significant increase in the percentage of CD4+ TCM as well as CD8+ TCM cells was observed in response to P8 (median/IQR in stimulated; unstimulated cultures for CD4+ and CD8+ TCM: 1.1/1.3; 0.3/0.1% and 0.3/0.3; 0.06/0.06), P13 (2/2; 0.4/0.8% and 0.5/1.3; 0.06/0.2), P14 (2/0.9; 0.3/0.1% and 0.3/0.4; 0.06/0.06) and P21 (2.3/1.4; 0.4/0.8% and 0.9/1.3; 0.06/0.2) (p≤0.04) (**Fig 11A and 11C**).

There was no significant change in the percentage of CD4+ TEM cells after peptide stimulation (**Fig 11B**).

The cured CL individuals exhibited high frequencies of both CD4+ TCM and TEM (median/IQR in stimulated; unstimulated cultures: 0.8/2; 0.3/0.9 and 59/15; 44/24) and CD8+ TCM and TEM cells (0.2/0.8; 0.05/0.2 and 43/28; 29/23), after SLA stimulation (p≤0.002) (**Fig 11**).

## Discussion

Vaccination is the best option to induce a successful host protective immunity against *Leishmania* parasites. There is currently no licensed vaccine for human use despite strong arguments supporting its feasibility. Peptide-based vaccines are composed by the most relevant synthetic short peptide fragments, making them safe, stable and easy to produce [40, 65–68].

Suitable human peptide-based vaccine candidates should contain both HLA-I and -II restricted epitopes, able to generate a properly balanced T cell response, since both CD4+ and CD8+ T cells are critical for mediating protective immune responses against *Leishmania* parasites.

In this study, we have selected three *Leishmania* proteins; H2B, PSA and LmlRAB for the prediction of HLA-I and -II binding peptides, using online available algorithms. These proteins were able to confer significant protection in mice and dogs [47–52, 69, 70]. However, these results should be interpreted with caution since the protection described in these animal models, which was induced against needle challenge with parasites, does not necessarily translate to protection following challenge by infected sand flies. Vaccine failure against infected sand fly challenge was associated with the inability of the vaccine to replicate the early kinetics of the secondary immunity observed in animals with a healed primary infection [71–73]. Nervertheless, we have previously reported that *Leishmania* H2B [49], *L*. *amazonensis* PSA protein (LaPSA-38S) [47] and LmlRAB [48], were able to induce a dominant Th1 profile of immune response in cured CL individuals, suggesting that these proteins are potential vaccine candidates against human leishmaniasis.

H2B, PSA and LmlRAB proteins were used to design eleven multi-epitope peptides composed of highly scored HLA-I or -II restricted epitopes, which were predicted to bind to at least 3/12 of the most common HLA-I and 3/5 of the most common HLA-II supertypes. It is interesting to note that, in combination, these multi-epitope peptides theoretically cover all HLA-I and -II supertypes analyzed, thus providing an optimal coverage in the human population. All peptides share a high to moderate level of sequence homology with different *Leishmania* species, a requisite for cross-species protection.

Most of the studies have focused on the identification of HLA-I epitopes [74–80] but only a few have been conducted to predict HLA-II [81–83] or a combination of both HLA-I and -II epitopes [23, 84, 85]. In this study, we described peptides containing multiple HLA-I and -II epitopes that would induce both CD8+ and CD4+ T cell responses, crucial for protection.

To confirm the ability of the predicted peptides to induce a specific immune response, we evaluated their immunogenicity in PBMC from individuals with healed CL due to *L*. *major*. It is well established that IFN-γ is a key effector cytokine, crucial to eliminate *Leishmania* parasites. Elevated levels of this cytokine are observed after *in vitro* stimulation, with *Leishmania* antigens, of PBMC from individuals who have developed a protective immunity against *L*. *major* [11, 86, 87]. We showed significant IFN-γ levels in response to pools containing HLA-I, HLA-II or HLA-I and -II multi-epitope peptides in cured CL individuals, whereas we could not detect any significant IFN-γ production in healthy subjects, indicating the *Leishmania* specificity of the responses observed. Furthermore, we noticed that most of the peptide pools containing both HLA-I and -II epitopes induced higher amounts of IFN-γ than those containing only HLA-I or -II epitopes. These results emphasize the importance to include both CD4+ and CD8+ specific T cell epitopes in peptide based vaccines [67, 88, 89].

Significant and specific levels of TNF-α, a cofactor for macrophage activation, were also observed in response to P21 peptide pool, in cured individuals. With regard to IL-10 production, we did not detect significant production in response to peptides used either individually or combined. Rather, some peptide pools induced a suppressive effect on IL-10 production. This anti-inflammatory cytokine has been involved in parasite persistence and disease establishment but also in controlling an excessive inflammatory response [15, 18, 90]. The immunomodulatory effect of some of our peptides may not be beneficial to the host, given the role of IL-10 in the regulation of exsessive IFN-γ responses [18]. A suppressive effect has also been reported in response to peptides derived from KMP-11 or GP63 proteins [40, 81].

These results suggest that multi-epitopes used as pools, can elicit a Th1-biased response, in individuals with healed CL. It is interesting to note that, among multi-epitope peptides used individually, H2BI and H2BII were able to induce significant levels of IFN-γ in cured CL individuals. In addition, all peptide pools, that were able to induce specific and significant IFN-γ, a part from P7, share HLA-I and/or HLA-II peptides derived from the H2B protein.

Some studies have shown significant levels of IFN-γ and low levels of IL-10 in response to HLA-I or HLA-II restricted epitopes derived from different *Leishmania* proteins, used alone or as pools, in cured CL or visceral leishmaniasis (VL) individuals [75, 77, 78, 83]. However, only a few studies have focused on the identification of both HLA-I and -II restricted peptides. Similar to our results, both highly promiscuous HLA-DR and HLA-I-binding epitopes, identified within nucleoside hydrolase NH36 domains, have been shown to induce IFN-γ production, though low, in asymptomatic DTH+ subjects [23]. Another study identified T cell epitopes with high affinity for both human HLA-I and -II within the proteome of *L*. *braziliensis*, which were able to stimulate the proliferation of lymphocytes from healed CL individuals, although the cytokine production was not evaluated [84]. Peptides including both highly scored murine MHC-I and II restricted epitopes derived from *L*. *infantum* proteins, induced high amounts of IFN-γ and low IL-10 levels in spleen cell culture supernatants from immunized mice [40]. More recently, it has been reported that recombinant chimeric proteins composed by human and murine MHC-I and II-specific T cell epitopes, were able to induce high IFN-γ/IL-10 ratios in both immunized mice and treated VL subjects [85, 91]. In addition, these recombinant chimeric proteins induced a protective immune response in vaccinated mice [85, 91]. On the other hand, some authors have identified HLA-I or -II-binding peptides that were unable to induce IFN-γ production in healed CL or VL subjects [76, 81]. The fact that synthetic epitopes alone are not immunogenic enough and the use of different culture conditions may explain these different results.

The reason we used multi-epitope peptides in our work is that these peptides are longer than isolated epitopes and would therefore be more immunogenic. Furthermore, a palmitoylated tail was added to our peptides, probably improving their immunogenicity [60, 61].

For further analysis, six peptide pools inducing the highest IFN-γ levels were selected. We validated their ability to activate specific IFN-γ producing cells in healed CL individuals, by ELISpot assay.

HLA typing was performed for some volunteers, for whom IFN-g positive responses were shown in response to peptide pools, thus validating the use of *in silico* methods for predicting epitopes in our proteins. However, a negative response was observed for one individual, probably due to the absence of HLA to which epitopes are able to bind or the absence of the predicted epitopes during natural infection. As previously reported for other antigens, not all of HLA binders lead to T cell stimulation [75, 92, 93].

Both CD4+ and CD8+ IFN-γ-secreting T cells responses are mandatory for *Leishmania* parasites elimination [10, 24, 26, 32, 36, 94]. An ideal vaccine candidate should therefore be able to induce both CD4+ and CD8+ T cell responses. We demonstrated that peptide pools were able to induce a significant increase of the percentages of both IFN-γ-producing CD4+ and CD8+ T cells (except P14), in cured individuals. These results suggest that the selected peptides include T cell epitopes that are naturally processed and presented to the immune system during infection. Some studies have shown that HLA-I or HLA-II restricted T cell epitopes derived from *L*. *major* or *L*. *donovani* proteins, used alone or as pools, were able to activate specific IFN-γ producing CD8+ or CD4+ T cells, in cured VL or CL subjects [75, 77, 78, 80, 82]. As far as we know, no study has described the detection of both cell populations in response to stimulation by both HLA-I and -II restricted peptides in humans. However, as we have described here, Agallou and colleagues designed multi-epitope peptides containing both MHC-I and -II restricted epitopes that were able to induce both IFN-γ-producing CD4+ and CD8+ T cells in immunized mice [40]. More interestingly, it was recently reported that mice vaccination with a chimera vaccine composed of F1 and F3 NH36 domains, holding respectively, epitopes involved in both CD8+ and CD4+ T cell mediated protective responses, was more efficient than vaccination with either of the domains injected separately [67, 89].

GrB, is a proapoptotic protease that has been associated with both tissue damage and good prognosis in CL and VL patients [28–32, 95, 96]. It was recently reported that GrB constitutes a good correlate for protection against severe forms of CL [33].

Although we did not detect GrB in response to P21 peptide pool by CBA, we showed a significant increase in the percentage of CD4+GrB+ T cells after stimulation with P8, P9, P16 and P21 peptide pools, in healed CL subjects. A high frequency of CD4+ T cells producing IFN-γ and GrB was also observed in response to P21 stimulation. Interestingly, we previously showed that LmlRAB protein, from which some of our peptides are derived, was able to induce significant GrB levels in healed CL individuals [48]. Significant levels of GrB in response to HLA-A*0201-restricted T cell epitopes from *L*. *major* excreted/secreted or to *Leishmania*-Activated C-Kinase (LACK) proteins and a high frequency of CD4+ T cells producing GrB in response to *L*. *major* excreted/secreted proteins, were reported in healed CL individuals [76, 87, 97]. The simultaneous expression of GrB and IFN-γ by CD8+ T cells was also described in cured patients [96].

The importance of multifunctional T cells that produce IFN-γ, TNF-α, and IL-2 in *Leishmania* infection control has been reported [20, 21, 23, 96]. Other authors suggested that multicytokine production may not be a requirement for optimal function of T effector cells that mediate concomitant immunity, in the context of a persisting primary infection [98]. In this study, the multiparameter cytometry analysis revealed that peptide pools P13, P14 and P21 were able to increase significantly the frequencies of bifunctional CD4+ IFN-γ+TNF-α+ and CD4+ IL-2+TNF-α+ T cells, confirming their ability to induce a specific Th1 response in healed CL subjects. However, no increases were observed in the frequencies of multifunctional CD4+ T cells producing simultaneously IFN-γ, TNF-α and IL-2. The induction of high proportions of both CD4+ and CD8+ IFN-γ+TNF-α+ by predicted T cell epitopes from NH36 or phosphoenolpyruvate carboxykinase (PEPCK) *Leishmania* proteins was described, respectively, in infected and vaccinated mice [67, 89, 99]. However, as far as we know, no study has described the detection of multifunctional T cells in response to stimulation by HLA restricted peptides in humans.

Finally, we showed that healed CL individuals exhibited a significant increase in the percentages of CD4+ and CD8+ TCM (CD45RO+CCR7+) cells when stimulated with P8, P13, P14 and P21 peptide pools. The period of *in vitro* lymphocyte culture used here (10 days) may explain the detection of central memory cells, rather than the more short-lived effector memory cells measured in *ex vivo* assays [100]. Moreover, the same peptide pools (except P13) also induced significant levels of IFN-γ, when analyzed by the cultured ELISPOT assay, which has been described to detect central memory T cells [101, 102]. It was demonstrated in a murine model of leishmaniasis that unlike TEM, TCM are maintained in the absence of parasites and mediate long-term resistance, suggesting that these cells should be the targets for nonlive vaccines against infectious diseases requiring cell-mediated immunity [103]. TCM cells were also identified in CL patients that have healed, suggesting that theses cells are generated during human leishmaniasis [35, 104]. However, it is important to emphasize that some observations in murine models of CL, based on the fact that the long-term persistence of parasites in the host is able to maintain resistance to reinfection (concomitant immunity), suggest that although TCM cells are essential components of protective immunity, they are probably not sufficient alone to induce optimal immunity required for protection against a sand fly-transmitted infection [98, 105, 106]. It has been shown that rapidly recruited, pre-existing CD4+Ly6C+T effector cells that are short-lived in the absence of infection, mediate concomittant immunity at the site of secondary infected sand fly challenge, in mice with a chronic primary infection [98, 107]. The authors suggest that the ability of a vaccine to induce an optimal protective immunity to *Leishmania* infection, may depend on the presence of both CD4+ TCM and T effector cells that should therefore be considered in vaccine development.

In this investigation, we have identified highly promiscuous HLA-I and -II restricted epitope combinations, derived from H2B, PSA and LmlRAB proteins, already involved in protection against *Leishmania* infection. These epitope combinations were able to induce IFN-γ-producing CD4+ and CD8+ T cells, GrB-producing CD4+ T cells, bifunctional CD4+ IFN-γ+TNF-α+ T cells, and CD4+ and CD8+ TCM cells, in individuals with healed CL. It is interesting to note that, among shorter peptide combinations, the P13 containing only three multi-epitope peptides composed of HLA-I and -II restricted epitopes from H2B and PSA proteins, was as efficient as P21 containing eleven multi-epitope peptides, in inducing CD4+ and CD8+ T cell responses. The data presented in this study represents a step forward in the identification of both CD4+ and CD8+ specific T cell epitopes that could be exploited for the design of a polytope vaccine against leishmaniasis.

## Supporting information

S1 Table HLA-typing of cured CL subjects and IFN-γ responses. (DOCX)

S1 Fig Gating strategy used to assess CD4+ T-cells producing IFN-γ or Granzyme B. (PDF)

S2 Fig Percentages of CD4+ T Cells producing IFN-γ and GrB to P21 peptide pool. (TIF)

S3 Fig Gating strategy used to assess CD8+ T-cells producing IFN-γ. (PDF)

S4 Fig Gating strategy used to assess multifunctional CD4+ T cells. (PDF)

S5 Fig Gating strategy used to assess CD4+ and CD8+ memory T cell. (PDF)
