# Supplementary material for: Design of multi-epitope peptides containing HLA class-I and class-II-restricted epitopes derived from immunogenic Leishmania proteins, and evaluation of CD4+ and CD8+ T cell responses induced in cured cutaneous leishmaniasis subjects
Source: PLoS Negl Trop Dis. 2020 Mar 16;14(3):e0008093. doi: 10.1371/journal.pntd.0008093 (PMC7098648; doi:10.1371/journal.pntd.0008093)
Supplement: S1 Table — (DOCX) [file pntd.0008093.s001.docx]

**S1 Table. HLA typing of cured CL subjects and IFN-γ responses.**

| **A- HLA typing** | | | | | |
| --- | --- | --- | --- | --- | --- |
| **Cured CL subjects** | **CCL58** | **CCL60** | **CCL61** | **CCL62** | **CCL64** |
| HLA-A | A*23:01  A*24:02 | A*03:01  A*24:01 | A*02:05  A*03:01 | A*24:02  A*29:02 | A*29:02  A*29:02 |
| HLA-B | B*08:01  B*49:01 | B*35:08  B*39:06 | B*35:08  B*49:01 | B*15:03  B*39:06 | B*35:01  B*49:01 |
| HLA-DRB | DRB1*07:01 or DRB1*07:79  DRB1*15:01 | DRB1*07:01 or DRB1*07:79 DRB1*11:01 | DRB1*11:01  DRB1*15:01 | DRB1*07:01 or DRB1* 07:79  DRB1*11:01 | DRB1*04:03  DRB1*11:01 |
| HLA-DPB | DPB*04:01  DPB1*17:01 | DPB1*13:01  DPB1*13:01 or DPB1*107:01 or DPB1*518:01 or DPB1*519:01 | DPB*04:01or DPB*350:01 or DPB1*702:01  DPB1*13:01 | DPB1*13:01  DPB1*17:01or DPB1*131:01 | DPB*04:01  DPB1*11:01 or DPB1*654:04 |
| **B- IFN-γ responses (Mean SFU/10^6^ PBMC±SD)** | | | | | |
| Unst1^a^ | 0±0 | 7±12 | 7±12 | 17±15 | 3±6 |
| PHA | 5895±1676 | 1095±205 | 5337±1072 | 3497±1158 | 3910±785 |
| SLA | 440±113 | 535±92 | 435±64 | 1817±242 | 1880±311 |
| Unst2^b^ | 10 ±0 | 7±6 | 0±0 | 0±0 | 3±6 |
| P8 | ND | 6550±679 | 1073±81 | 1565±106 | 890±111 |
| P9 | 1620±820 | 1405±488 | 43±75 | 10±14 | 7±6 |
| P16 | 2303±267 | 1765±7 | 10±0 | 207±112 | 290±105 |
| P21 | 1560±495 | 815±177 | 4790±2927 | 87±40 | 410±177 |

^a^Unstimulated cultures, negative control for PHA and SLA-stimulated cultures

^b^Unstimulated cultures, negative control for peptide pools-stimulated cultures
