# Supplementary material for: Design of multi-epitope peptides containing HLA class-I and class-II-restricted epitopes derived from immunogenic Leishmania proteins, and evaluation of CD4+ and CD8+ T cell responses induced in cured cutaneous leishmaniasis subjects
Source: PLoS Negl Trop Dis. 2020 Mar 16;14(3):e0008093. doi: 10.1371/journal.pntd.0008093 (PMC7098648; doi:10.1371/journal.pntd.0008093)
Supplement: S3 Fig — (PDF) [file pntd.0008093.s004.pdf]

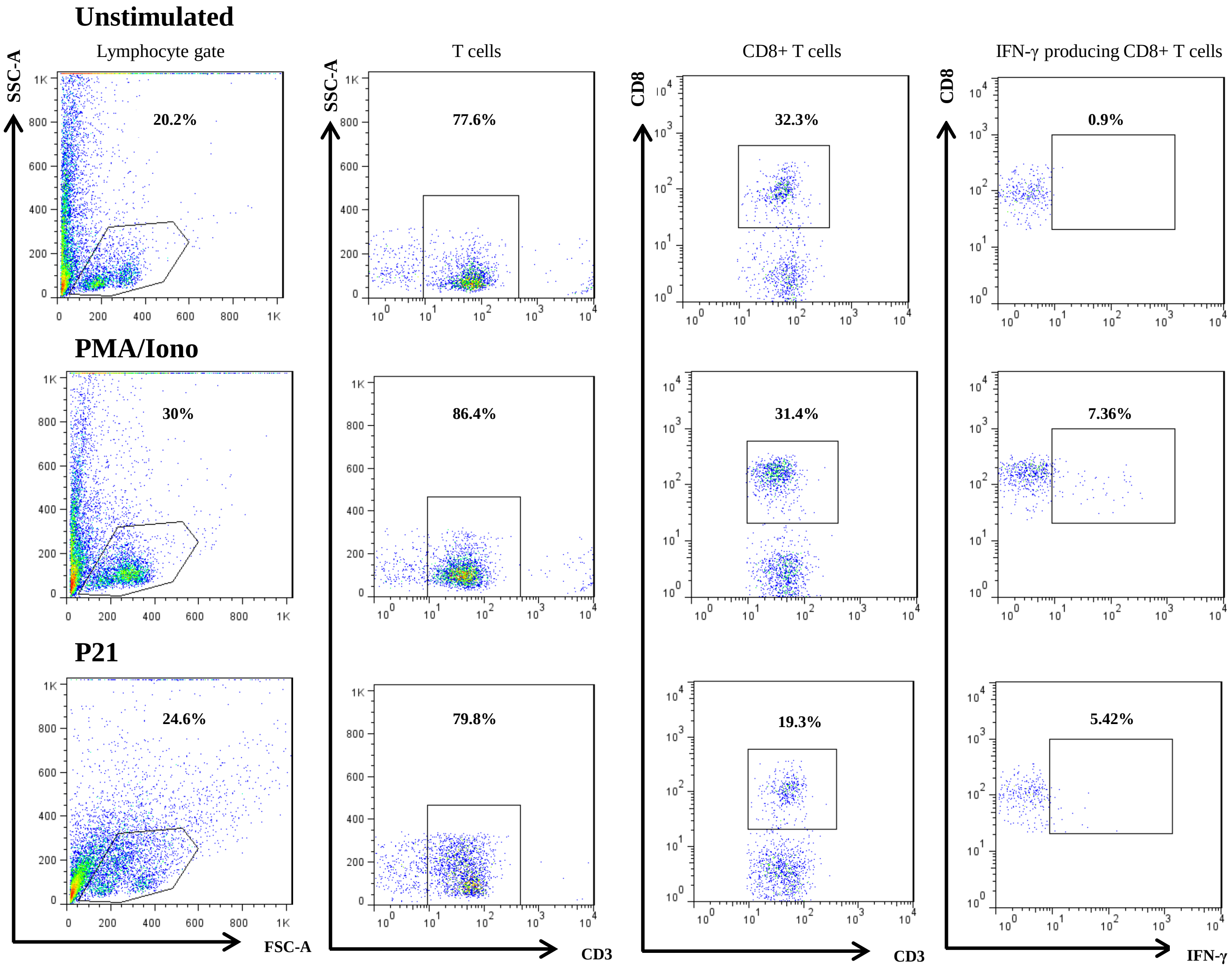

S3 Fig. Gating strategy used to assess CD8+ T-cells producing IFN-γ

Representative dot plots from one cured CL individual. Lymphocytes were identified and gated according to FSC-A (size) vs SSC-A (granularity). T cells were then distinguished by the CD3 expression. CD8+ T cells were identified by CD3 and CD8 expression. The CD8+ T cells gates were further analyzed for IFN-γ expression.
