# Supplementary material for: Design of multi-epitope peptides containing HLA class-I and class-II-restricted epitopes derived from immunogenic Leishmania proteins, and evaluation of CD4+ and CD8+ T cell responses induced in cured cutaneous leishmaniasis subjects
Source: PLoS Negl Trop Dis. 2020 Mar 16;14(3):e0008093. doi: 10.1371/journal.pntd.0008093 (PMC7098648; doi:10.1371/journal.pntd.0008093)
Supplement: S4 Fig — (PDF) [file pntd.0008093.s005.pdf]

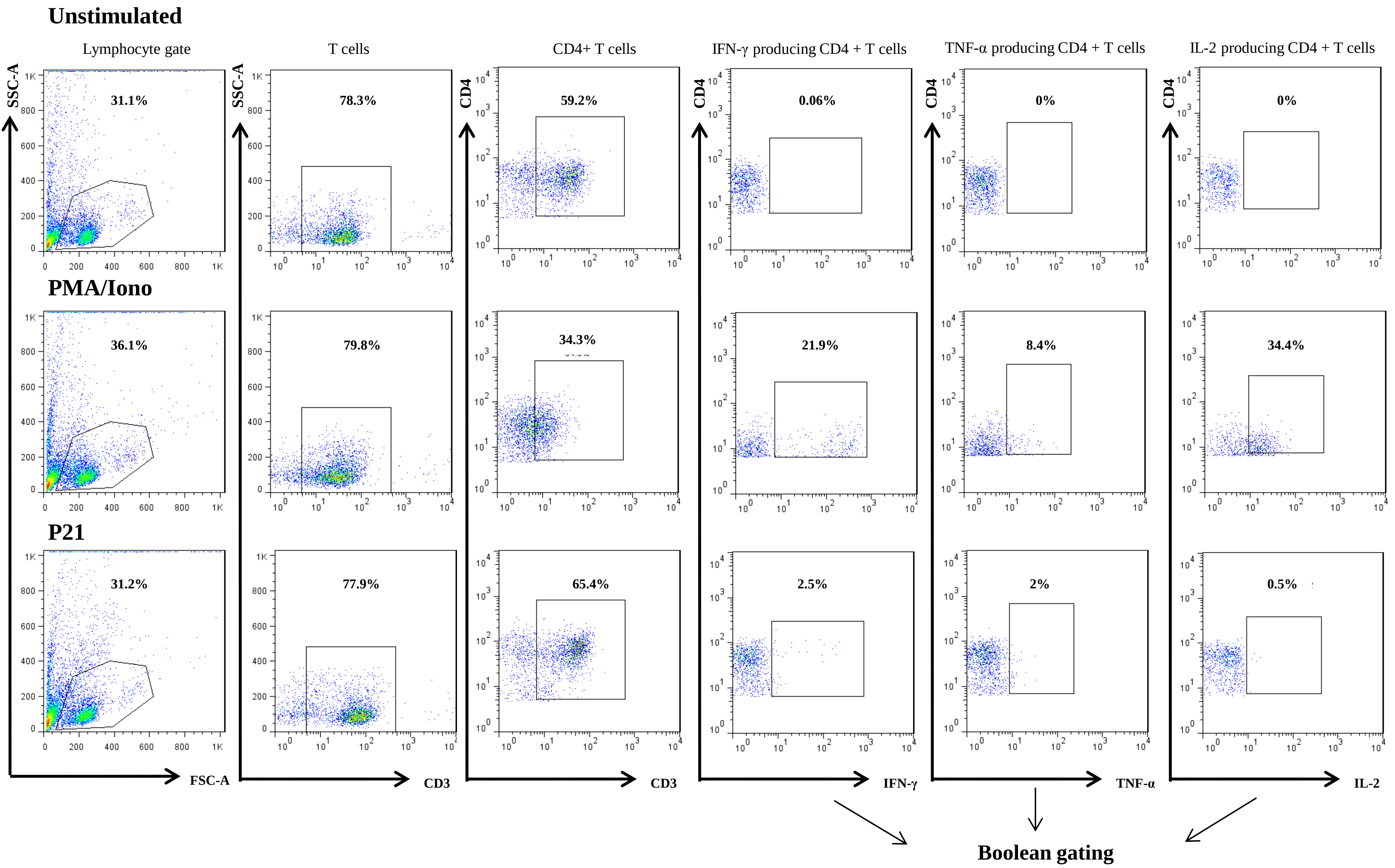

S4 Fig. Gating strategy used to assess multifunctional CD4+ T cells

Representative dot plots from one cured CL individual. Lymphocytes were identified and gated according to FSC-A (size) vs SSC-A (granularity), followed by CD3+ gating. CD4+ T cells were identified by CD3 and CD4 expression. Antigen-specific IFN-γ-, IL-2- and TNF-α-producing CD4+ T cells were gated as shown. Boolean gating (using the Boolean operations 'and' and 'not') of the three cytokines gates (IFN-γ, IL-2 and TNF-α) was performed to determine the frequencies of all possible combinations of cytokine-producing CD4+ T cells using FlowJo software.
